# Oral nitrate supplementation improves cardiovascular risk markers in COPD: ON-BC, a randomised controlled trial

**DOI:** 10.1183/13993003.02353-2022

**Published:** 2024-02-01

**Authors:** Ali M. Alasmari, Abdullah S. Alsulayyim, Saeed M. Alghamdi, Keir E.J. Philip, Sara C. Buttery, Winston A.S. Banya, Michael I. Polkey, Paul C. Armstrong, Matthew J. Rickman, Timothy D. Warner, Jane A. Mitchell, Nicholas S. Hopkinson

**Affiliations:** 1 National Heart and Lung Institute, Royal Brompton Campus, Imperial College London, London, UK; 2 Respiratory Therapy Department, College of Medical Rehabilitation Sciences, Taibah University, Madinah, Saudi Arabia; 3 Respiratory Therapy Department, Faculty of Applied Medical Sciences, Jazan University, Jazan, Saudi Arabia; 4 Clinical Technology Department, Umm Al-Qura University, Makkah, Saudi Arabia; 5 Respiratory Medicine, Royal Brompton and Harefield Hospitals, London, UK; 6 Centre for Immunobiology, Blizard Institute, Faculty of Medicine and Dentistry, Queen Mary University of London, London, UK; 7 National Heart and Lung Institute, Cardiothoracic Pharmacology, Vascular Biology, Imperial College London, London, UK

## Abstract

**Background:**

Short-term studies suggest that dietary nitrate (NO_3_^−^) supplementation may improve the cardiovascular risk profile, lowering blood pressure (BP) and enhancing endothelial function. It is not clear if these beneficial effects are sustained and whether they apply in people with COPD, who have a worse cardiovascular profile than those without COPD. Nitrate-rich beetroot juice (NR-BRJ) is a convenient dietary source of nitrate.

**Methods:**

The ON-BC trial was a randomised, double-blind, placebo-controlled parallel group study in stable COPD patients with home systolic BP (SBP) measurement ≥130 mmHg. Participants were randomly allocated (1:1) using computer-generated, block randomisation to either 70 mL NR-BRJ (400 mg NO_3_^−^) (n=40) or an otherwise identical nitrate-depleted placebo juice (0 mg NO_3_^−^) (n=41), once daily for 12 weeks. The primary end-point was between-group change in home SBP measurement. Secondary outcomes included change in 6-min walk distance (6MWD) and measures of endothelial function (reactive hyperaemia index (RHI) and augmentation index normalised to a heart rate of 75 beats·min^−1^ (AIx75)) using an EndoPAT device. Plasma nitrate and platelet function were also measured.

**Results:**

Compared with placebo, active treatment lowered SBP (Hodges–Lehmann treatment effect −4.5 (95% CI −5.9– −3.0) mmHg), and improved 6MWD (30.0 (95% CI 15.7–44.2) m; p<0.001), RHI (0.34 (95% CI 0.03–0.63); p=0.03) and AIx75 (−7.61% (95% CI −14.3– −0.95%); p=0.026).

**Conclusions:**

In people with COPD, prolonged dietary nitrate supplementation in the form of beetroot juice produces a sustained reduction in BP, associated with an improvement in endothelial function and exercise capacity.

## Tweetable abstract

**The ON-BC trial shows that 12 weeks of oral nitrate supplementation, in the form of beetroot juice, lowers blood pressure, improves vascular function and increases exercise capacity in people with COPD** https://bit.ly/3MAGiZM

## Introduction

COPD, a common condition associated with substantial morbidity and mortality, is the third leading cause of death worldwide [1]. Although COPD is a lung condition, it is associated with significant extrapulmonary pathology, in particular cardiovascular disease [2]. The mechanisms driving this are complex, being influenced by a number of shared pathophysiological mechanisms including smoking, systemic cellular inflammation, oxidative stress and sedentarism [3–6].

Nitric oxide (NO) plays a pivotal role as a vasodilator modulating vascular tone, blood pressure (BP) and haemodynamics as well as influencing the oxygen cost of exercise [7]. Dietary nitrate (NO_3_^−^) supplementation using nitrate-rich beetroot juice (NR-BRJ) has received growing attention in cardiovascular and exercise research [8], although clinical data are scarce and studies to date have mostly been of short duration [9–14]. There has also been interest in the effect of beetroot juice in people with COPD because of potential benefits on exercise capacity [15], particularly in hypoxic individuals [16], and in the context of pulmonary rehabilitation [17].

However, beetroot juice might also improve the cardiovascular risk profile in this population who are at high risk of cardiovascular events [2, 18]. A recent systematic review investigating the effect of dietary nitrate in chronic respiratory disease set out the need for longer term, adequately powered studies to establish if short-term beneficial effects persist [19]. We therefore investigated the hypothesis that in people with COPD with systolic BP (SBP) >130 mmHg (at least “high normal”) [20], a once-daily dose of (70 mL) NR-BRJ for 12 weeks would produce a sustained fall in BP compared with placebo, accompanied by improvements in endothelial function and exercise capacity.

## Methods

The Oral Nitrate for Blood pressure in COPD (ON-BC) study was a prospective, double-blind, parallel group, randomised, placebo-controlled trial. The study was conducted in accordance with the Declaration of Helsinki and was approved by the London – West London & GTAC Research Ethics Committee (19/LO/1660). The trial was prospectively registered at the ISRCTN registry with identifier ISRCTN47839214.

Further details of the methods are available in the supplementary material.

### Participants

**FIGURE 1:**
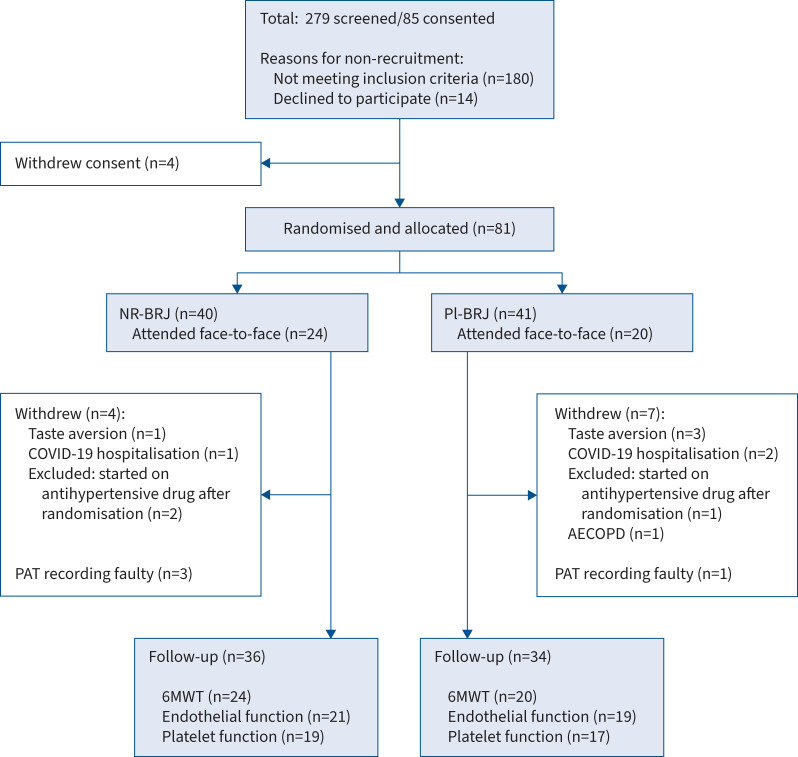
CONSORT flow diagram for the Oral Nitrate for Blood pressure in COPD (ON-BC) trial. NR-BRJ: nitrate-rich beetroot juice; Pl-BRJ: placebo beetroot juice; COVID-19: coronavirus disease 2019; AECOPD: acute exacerbation of COPD; PAT: peripheral arterial tonometry; 6MWT: 6-min walk test.

All participants provided written informed consent before enrolment in the study. We recruited people with COPD from clinics at the Royal Brompton Hospital (London, UK) between October 2020 and August 2021 as outlined in the participant flow diagram in figure 1. To be eligible, participants had to have stable COPD without an acute exacerbation or change in medication in the preceding month and have a baseline SBP ≥130 mmHg [20]. Exclusion criteria included a diagnosis of significant comorbidity limiting life expectancy below the duration of the study, use of more than three antihypertensive medications or use of nitrate-based medications.

### Study procedure and intervention

Participants were allocated randomly to active treatment or placebo (1:1) using an online randomisation system (Sealed Envelope; www.sealedenvelope.com) stratified by whether or not they were on antihypertensive medication. In the active arm, participants received NR-BRJ (70 mL Beet It Sport Nitrate Shot; James White Drinks, Ipswich, UK) which contains 400 mg NO_3_^−^. The placebo group received a nitrate-depleted, but otherwise identical in taste and colour, placebo beetroot juice (Pl-BRJ) produced by the same company [16]. Participants were asked to drink their intervention juice, one beverage daily at 09:00, for 90 days. The trial intervention was delivered directly to participants' homes by the manufacturer. Participants were asked not to alter their usual diet and to keep antihypertensive medication(s) constant (in treated patients with hypertension). Study procedures are outlined in supplementary figure S1.

### Outcome measures

The primary outcome was change in SBP, assessed using a validated home BP upper arm monitor (Omron M3 Comfort (HEM-7134-E); Omron Healthcare, Kyoto, Japan). Participants recorded two BP measurements, three times daily over 4 days at baseline and then again during the last week of the study. Day 1 values were discarded and the BP values used for analyses were taken as a mean of all measures recorded on days 2–4 during the assessment period.

### COVID adaptation

Because of the coronavirus disease 2019 pandemic, some participants were unable to or chose not to attend for face-to-face study visits. The study was modified to allow the primary and secondary end-points (quality of life (QoL) questionnaires) to be collected remotely where this was possible. BP log sheets, juice adherence log sheets, the COPD Assessment Test (CAT) [21] used for QoL assessment and the Medical Research Council (MRC) dyspnoea scale used for breathlessness assessment were mailed to remote participants, including a reply-paid envelope for return. Telephone calls were made at 6 and 11 weeks to encourage compliance, collect information on adverse events, promote home BP monitoring and encourage the return of trial outcome data. Only those attending for face-to-face visits had a 6-min walk test performed according to American Thoracic Society guidelines [22] as a secondary end-point, as well as blood tests and measures of vascular function as exploratory end-points.

### Vascular function

Arterial stiffness and endothelial function were assessed non-invasively using finger plethysmography peripheral arterial tonometry (EndoPAT2000; Itamar Medical, Caesarea, Israel), which provides the reactive hyperaemia index (RHI) score as well as measuring the augmentation index normalised to a heart rate of 75 beats·min^−1^ (AIx75). A lower RHI is associated with endothelial dysfunction, while a higher AIx75 is a marker of vascular stiffness (supplementary material) [23–27].

### Biomarkers

Blood samples were obtained to measure renal function and brain natriuretic peptide (BNP) levels. Plasma nitrogen oxides (NO*_x_*) concentrations were determined using a commercial colorimetric assay (Nitrate/Nitrite Colorimetric Assay Kit 780001; Cayman Chemicals, Ann Arbor, MI, USA) based on the Griess method as described previously [28].

To assess the effect of study intervention on platelet reactivity (platelet aggregation and P-selectin), blood was pipetted (40 µL) into 96-well plates pre-coated with platelet agonists or vehicle. Plates were then placed on a plate shaker (BioShake iQ; QInstruments, Jena, Germany) for 5 min at 1000 rpm and 37°C, after which samples were fixed by addition of 160 µL pre-prepared acid citrate dextrose to each well and plates stored at 4°C. Levels of platelet activation in samples were analysed within 24 h by flow cytometry [29]. For these analyses, we excluded participants who were on antiplatelet therapy.

Exhaled NO fraction (*F*_ENO_) was measured using the NIOX VERO device (Circassia, London, UK). Both study participants and researchers were kept blind to intervention allocation, outcome measurements and the results. Data were uploaded into a password-protected database by an independent researcher who was not otherwise involved in the trial.

### Statistical analysis and sample size calculation

The ON-BC trial was powered for the primary outcome change in home SBP response at 12 weeks from baseline. Prior data from our group in the context of pulmonary rehabilitation showed a fall with supplementation of 5±3.7 mmHg [17]. Taking this standard deviation, to identify a 3 mmHg difference in fall in SBP, with 90% statistical power at a significance level of 0.05, would require 32 participants in each arm to complete the study. Allowing for a 10% withdrawal rate, a sample size of 72 participants would be required. Data analysis was performed on an intention-to-treat basis.

Categorical data are presented as number (percentage) and comparisons between the groups performed using the Chi-squared or Fisher's exact test. Numerical data are presented as mean with standard deviation or as median (interquartile range (IQR)) depending on the distribution of the data (based on the Shapiro–Wilk test for normality). The treatment effect was estimated by subtracting the baseline values from the 12-week values, then using a two-sample independent t-test or the Wilcoxon rank-sum test to compare the groups. Where the t-test was used the treatment effect was reported as the difference between the two means with 95% confidence intervals and where the Wilcoxon rank-sum test was used the treatment effect was estimated with the Hodges–Lehmann estimate. The Hodges–Lehmann process entails estimating the average difference in outcomes (*x*–*y*) for every possible *n*(*n*+1)/2 pair and then deriving the overall median of all averages (the Hodges–Lehmann estimator). A distribution-free confidence interval is estimated using large-sample approximation. Analyses were performed using SPSS for Windows version 27.0 (IBM, Armonk, NY, USA) and Prism version 69.0 for Mac (GraphPad, San Diego, CA, USA). A p-value of <0.05 was considered to be statistically significant.

## Results

### Study participants

From 279 individuals screened for eligibility, 81 participants were randomised to receive Pl-BRJ (n=41) or NR-BRJ (n=40), of whom 70 (Pl-BRJ n=34 and NR-BRJ n=36) completed the study protocol and had complete data. 44 participants (Pl-BRJ n=20 *versus* NR-BRJ n=24) attended face-to-face visits and in 37 the study was conducted remotely. Participants in the two study arms were well matched for baseline characteristics, particularly for SBP (Pl-BRJ 136 (132–140) mmHg *versus* NR-BRJ 135 (130–143) mmHg) and use of antihypertensive medications (17 *versus* 15, respectively; p=0.477) (table 1). Most participants were of Caucasian ethnicity, except for two of Afro-Caribbean and one of Asian ethnicity.

**TABLE 1:** Demographic and baseline characteristics

	**Pl-BRJ (n=34)**	**NR-BRJ (n=36)**	**p-value**
**Male**	22 (64.7)	26 (72.2)	0.50
**Age (years)**	64.5±7.4	62.5±7.4	0.33
**Ethnicity**			
** **White Caucasian	33 (97.1)	34 (94.4)	0.49
** **Black	0	2 (5.6)	
** **Asian	1 (2.9)	0	
**BMI (kg·m^−2^)**	26 (24–30)	27 (23.3–30)	0.81
**Smoking status**			
** **Ex-smoker	30 (88.2)	30 (83.3)	0.74
** **Current smoker	4 (11.8)	6 (16.7)	
** **Smoking history (pack-years)	32 (30–40)	30 (20–40)	0.63
**FEV_1_ % pred**	39.6±15.2	45.2±14.9	0.12
**Cardiovascular comorbidities**			
** **Hypercholesterolaemia	7 (20.6)	9 (25.0)	0.66
** **Hypertension	17 (50.0)	15 (41.7)	0.48
** **Ischaemic heart disease	6 (17.7)	8 (22.2)	0.63
**Antihypertensive medications**			
** **Nil	17 (50.0)	21 (58.3)	0.48
** **Single medication	15 (44.1)	11 (30.6)	0.24
** **Two medications	2 (5.6)	4 (11.1)	0.44
** **ACEi/ARBs	10 (29.4)	9 (25.0)	0.68
** **CCBs	4 (11.8)	8 (22.2)	0.35
** **β-blockers	3 (8.8)	7 (19.4)	0.31
** **Diuretics	2 (5.6)	7 (19.4)	0.15
**Mouthwash use**			
** **1–3 times per week	9 (26.5)	9 (25.0)	0.95
** **4–7 times per week	6 (17.6)	5 (13.9)	
**Baseline home BP, mmHg**			
** **SBP	136 (132–140)	135 (130–143)	0.45
** **DBP	85.7±5.1	85.0±7.6	0.65
** **MAP	103 (100–105)	102.5 (98–106.5)	0.85
**Baseline endothelial function markers**			
** **Endothelial RHI score (n=19 *versus* 21)	2.11±0.50	2.04±0.41	0.68
** **AIx75 (%) (n=19 *versus* 21)	18.6 (13.0–35.1)	22.8 (14–29.5)	0.46
**eGFR (mL·min^−1^·1.73 m^−2^** **) (n=20 *versus* 24)**	90 (80.5–90)	83 (72–90)	0.07
**BNP (ng·L^−1^) (n=20 *versus* 24)**	36.5 (26–50)	27.5 (18.5–43.5)	0.14
**NO*_x_*** **(μmol·L^−1^) (n=20 *versus* 24)**	36.9 (29.9–46.3)	29.3 (25.0–45.4)	0.27

Data are presented as n (%), median (interquartile range) or mean±sd. Pl-BRJ: placebo beetroot juice; NR-BRJ: nitrate-rich beetroot juice; BMI: body mass index; FEV_1_: forced expiratory volume in 1 s; ACEi: angiotensin-converting enzyme inhibitor; ARBs: angiotensin receptor blocker; CCB: calcium channel blocker; BP: blood pressure; SBP: systolic BP; DBP: diastolic BP; MAP: mean arterial pressure (calculated as MAP∼(SBP+(DBP×2))/3); RHI: reactive hyperaemia index; AIx75: augmentation index normalised to a heart rate of 75 beats·min^−1^; eGFR: estimated glomerular filtration rate; BNP: brain natriuretic peptide; NO*_x_*: nitrogen oxides.

### Intervention tolerance and compliance

The intervention was generally well tolerated by both groups. Two participants from the placebo group and one from the active group withdrew from the study in the first week because they found the juice unpalatable. Otherwise, there were high rates of compliance assessed through diary card returns (Pl-BRJ 96% *versus* NR-BRJ 97%; p=0.78). No serious adverse events were reported. Five participants (NR-BRJ n=3 and Pl-BRJ n=2) reported discolouration of their urine (beeturia).

### Effect of dietary nitrate supplementation on BP

**FIGURE 2:**
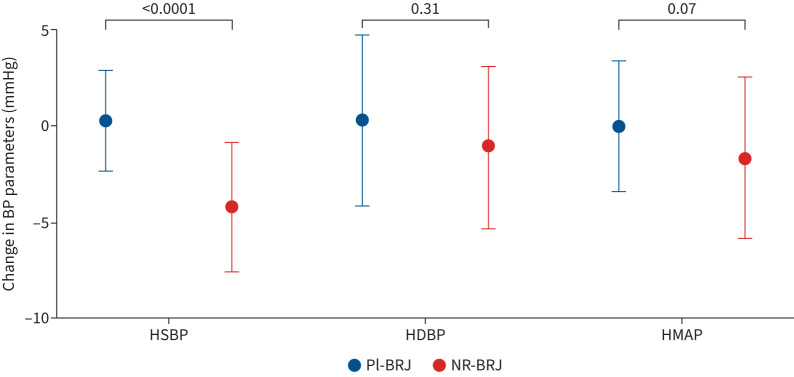
Effect of dietary nitrate supplementation on blood pressure (BP) parameters. Change in home-monitored BP parameters in placebo beetroot juice (Pl-BRJ; n=34) *versus* nitrate-rich beetroot juice (NR-BRJ; n=36) subjects with COPD. Data are presented as mean±sd. HSBP: home systolic BP; HDBP: home diastolic BP; HMAP: home mean arterial pressure (calculated as MAP∼(SBP+(DBP×2))/3).

Dietary nitrate supplementation was associated with a fall in SBP compared with placebo (Hodges–Lehmann treatment effect −4.5 (95% CI −3.0– −5.9) mmHg; p<0.001) (figure 2 and table 2) and in mean arterial pressure (−1.67 (95% CI −1.81–0.04) mmHg; p=0.07). There was no statistically significant difference in diastolic BP (−1.1 (95% CI −3.1–1.0) mmHg; p=0.31).

**TABLE 2:** Impact of dietary supplementation on study outcomes

	**Pl-BRJ (n=34)**			**NR-BRJ (n=36)**			**Treatment effect^#^ (95% CI)**	**p-value**
	**Pre**	**Post**	**Difference**	**Pre**	**Post**	**Difference**		
**HSBP (mmHg)**	136 (132–140)	137 (132–140)	0.3±2.6	135 (130–143)	131 (127–136)	−4.2±3.3	−4.5 (−5.9– −3.0)	<0.0001
**HDBP (mmHg)**	85.7±5.1	85.9±7.4	0.2±4.4	85.0±7.6	84.2±8.0	−0.4±4.2	−1.1 (−3.1–1.0)	0.31
**HMAP (mmHg)**	103 (100–105)	103 (99–106)	−0.03±3.4	102.5 (98–106.5)	102 (94–106.5)	−1.7±4.2	−1.67 (−1.81–0.04)	0.07
**Heart rate (beats·min^−1^)**	77.7±10.8	79.5±10.8	1.8±4.2	77.3±9.4	78.1±10.1	0.8±4.9	−1.0 (−3.1–1.3)	0.30
**6MWD (m)**	362.6±91.4	355.6±98.0	−7.0±21.8	384.5±74.8	407.5±72.4	23.0±24.9	30.0 (15.7–44.2)	<0.0001
**CAT score**	21.4±7.1	20.9±7.1	−0.5±3.4	21.9±7.2	20.3±6.9	−1.6±4.2	−1.1 (−3.0–0.7)	0.22
***F*_ENO_ (ppb)**	17 (15–24.5)	22 (18–26)	5 (−1–8.5)	19 (12–36)	30 (20.5–55)	11 (5–24)	9 (2–17)	0.012
**RHI score**	2.11±0.50	1.81±0.49	−0.3±0.46	2.04±0.41	2.08±0.50	0.04±0.48	0.34 (0.03–0.63)	0.03
**AIx75 (%)**	18.6 (13.0–35.1)	18 (13.1–31.5)	0.15±12.22	22.8 (14.0–29.5)	14.0 (11.3–22.0)	−7.46±8.41	−7.61 (−14.27– −0.95)	0.026
**BNP (ng·L^−1^)**	36.5 (26–50)	35 (26–45)	−0.4±16.1	27.5 (18.5–43.5)	36 (15.5–45)	−0.7±19.1	−0.3 (−11.3–10.7)	0.68
**eGFR (mL·min^−1^·1.73 m^−2^)**	90 (80.5–90)	88 (83.5–90)	0.20±7.00	83 (72–90)	82 (65–90)	0.63±5.32	−0.83 (−4.57–2.92)	0.66
**Creatinine (µmol·L^−1^)**	70 (58–82)	71 (59–77)	0.16±6.50	73 (64–91.5)	77 (67–97.5)	2.54±8.54	2.38 (−2.40–7.17)	0.32
**Urea (mmol·L^−1^)**	4.9 (4.3–6.3)	5.0 (4.0–6.1)	−0.15±1.20	5.4 (4.25–6.75)	5.1 (4.2–6.25)	0.11±1.18	0.26 (−0.47–1.01)	0.47
**NO*_x_*** **(μmol·L^−1^)**	36.9 (29.9–46.3)	32.6 (27.5–45.1)	−2.25 (−9.7–7.3)	29.3 (25.0–45.4)	351.8 (265.3–524.8)	318.3 (237.8–460.8)	319 (279.5–397.3)	<0.0001

Categorical data are presented as n (%) and comparisons between the groups done using the Chi-squared or Fishers exact test; numerical data are presented as mean±sd or median (interquartile range) depending on the distribution of the data. Pl-BRJ: placebo beetroot juice; NR-BRJ: nitrate-rich beetroot juice; HSBP: home systolic blood pressure; HDBP: home diastolic blood pressure; HMAP: home mean arterial pressure (calculated as MAP∼(SBP+(DBP×2))/3); 6MWD: 6-min walk distance; CAT: COPD Assessment Test; *F*_ENO_: exhaled nitric oxide fraction; RHI: reactive hyperaemia index; AIx75: augmentation index normalised to a heart rate of 75 beats·min^−1^; BNP: brain natriuretic peptide; eGFR: estimated glomerular filtration rate; NO*_x_*: nitrogen oxides. ^#^: the treatment effect was estimated by subtracting the baseline values from the 12-week values, then using a two-sample independent t-test or the Wilcoxon rank-sum test to compare the groups. Where the t-test was used the treatment effect was reported as the difference between the two means with 95% confidence intervals and where the Wilcoxon rank-sum test was used the treatment effect was estimated with the Hodges–Lehmann estimate. The Hodges–Lehmann process entails estimating the average difference in outcomes (*x*–*y*) for every possible *n*(*n*+1)/2 pair and then deriving the overall median of all averages (the Hodges–Lehmann estimator). A distribution-free confidence interval is estimated using large-sample approximation.

### Exercise capacity and symptoms

**FIGURE 3:**
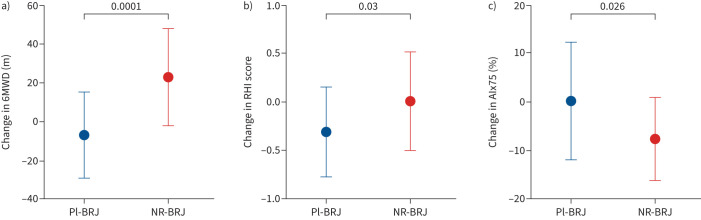
Effect of dietary nitrate supplementation on exercise capacity and endothelial function. a) 6-min walk distance (6MWD) in placebo beetroot juice (Pl-BRJ; n=20) and nitrate-rich beetroot juice (NR-BRJ; n=24) COPD individuals. b, c) Endothelial function assessed, in Pl-BR (n=19) compared with NR-BRJ (n=21), as b) reactive hyperaemia index (RHI) and c) augmentation index normalised to a heart rate of 75 beats·min^−1^ (AIx75). Data are presented as mean±sd.

Among participants attending face-to-face (Pl-BRJ n=20 and NR-BRJ n=24), there was an improvement in 6MWT distance associated with active treatment (30.0 (95% CI 15.7–44.2) m; p<0.001) (figure 3). No statistically significant between-group effects were observed for the CAT score (−1 (95% CI −3–0); p=0.09) or the MRC dyspnoea score (0 (95% CI −1–1); p=0.46).

### Endothelial function

Paired measures were available for 40 participants (three participants had poor recording signals and one participant had a finger deformity). Dietary nitrate ingestion was associated with improvements in both the endothelial RHI score (Hodges–Lehmann treatment effect 0.34 (95% CI 0.03–0.63); p=0.03) and AIx75% (−7.61% (95% CI −14.3– −0.95); p=0.026) compared with placebo (figure 3).

There was no difference observed in change in BNP levels between groups nor in markers of renal function (estimated glomerular filtration rate, creatinine and urea) following active treatment compared with placebo (table 2).

### Plasma NO*_x_* concentrations and *F*_ENO_ levels

**FIGURE 4:**
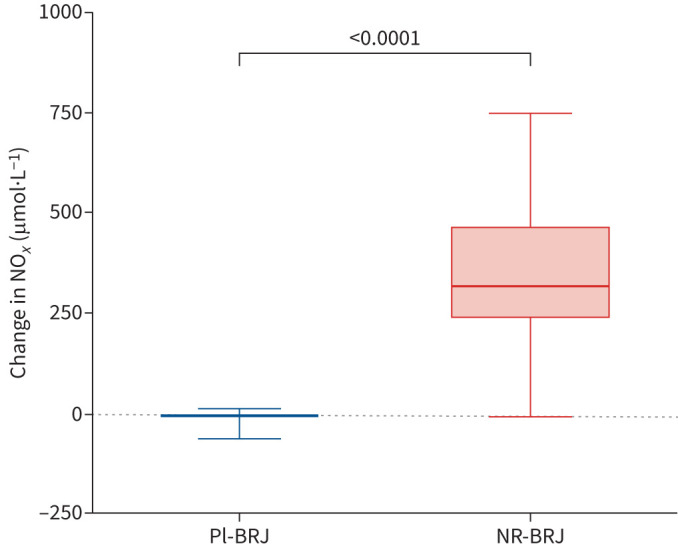
Effect of dietary nitrate supplementation on plasma nitrogen oxides (NO*_x_*) concentrations in individuals with COPD. Data are presented as median (interquartile range); whiskers indicate minimum–maximum range. Pl-BRJ: placebo beetroot juice; NR-BRJ: nitrate-rich beetroot juice.

Data on plasma NO*_x_* concentrations were available for 44 study participants (Pl-BRJ n=20 and NR-BRJ n=24). As expected, we observed a substantial increase in plasma NO*_x_* from baseline in the active treatment group but not in the placebo group (table 2 and figure 4). We measured *F*_ENO_ in 44 face-to-face participants There was a statistically significant increase in *F*_ENO_ levels associated with consumption of NR-BRJ compared with Pl-BRJ (mean difference 12.0 (95% CI 3.57–20.5) ppb; p=0.01).

### Platelet aggregation

12 weeks of NR-BRJ ingestion did not lead to any changes in platelet aggregation compared with placebo for arachidonic acid (−1.2% (95% CI −13–12%); p=0.85), collagen (9.5% (95% CI −8.9–28%); p=0.30), TRAP6-amide (11% (95% CI −2.0–24%); p=0.09) or U46619 (0.44% (95% CI −13–14%); p=0.94), respectively (supplementary figure S4). Similarly, no differences were seen in P-selectin expression (1.7% (95% CI −5.1–8.5%); p=0.63) (supplementary figure S5).

### Impact of mouthwash use

Nitrate is reduced to nitrite by bacteria in the mouth. Use of mouthwash, which can influence the oral microbiome, might therefore attenuate the effect of dietary nitrate supplementation. However, use did not differ between study arms, being reported by 15 participants (44%) in the Pl-BRJ arm and 14 participants (39%) in the NR-BRJ arm, with six and five participants, respectively, using an antiseptic product, the others a fluoride product. In a post-hoc analysis using a mixed effects model we found no evidence of an interaction between mouthwash use and treatment effect.

## Discussion

The main finding of the ON-BC trial is that dietary nitrate supplementation, in the form of beetroot juice, produces a sustained fall in SBP in people with COPD which is accompanied by improvements in exercise capacity and markers of endothelial function.

### Significance of findings

Although previous short-term studies have suggested potential beneficial effects of beetroot juice as a form of dietary nitrate supplementation in healthy individuals, primary hypertensive and untreated hypercholesteraemic patients [10, 12, 19, 30, 31], and one 6-month study has shown an improvement in central but not peripheral SBP in people with or at risk from type 2 diabetes [14], the present trial, over 12 weeks, is the longest duration study of this intervention in COPD. Our findings are also broadly consistent with the largest randomised controlled trial of dietary nitrate supplementation in COPD (the ON-EPIC study), which demonstrated a fall in SBP of 5±3.7 mmHg with dietary nitrate supplementation over 8 weeks in people with COPD, conducted in the context of a pulmonary rehabilitation programme [17]. A 5-week study of nitrate supplementation did not show any reduction in BP in people with hypertension, but this lack of effect may have been due to the use of a lower nitrate dose (300 mg) than was used in the present study [13]. The fall in BP observed in this study is sufficient to impact on the primary and secondary prevention of cardiovascular events in people with COPD (and potentially other conditions). Findings reported in 1997 by Stamler [32] from the INTERSALT epidemiological study, which included results of BP parameters from more than 10 000 men and women, suggested that a 3 mmHg reduction of SBP in the population would result in an 8% overall reduction in mortality due to a stroke, a 5% reduction in mortality due to coronary heart disease and a 4% decrease in all-cause mortality. More recently, a meta-analysis of 123 studies including 613 815 participants showed a continual reduction in risk from lowering BP even into what have been considered to be normal BP ranges, which is particularly relevant in increased risk populations, including people with COPD [33]. Importantly, people with COPD have increased vascular risk over and above that accorded by standard risk scores [2, 18].

Although the mechanism of action of NO_3_^−^ in reducing BP is currently unclear, it is likely due to the endogenous sequential reduction of the nitrate–nitrite–NO pathway by various enzymatic and non-enzymatic reductases stimulating the vasodilatation [34]. In this study, the substantial increases in plasma NO*_x_* concentration after NR-BRJ were associated with SBP reduction, providing further evidence that nitrate/nitrite reduction to NO most likely underlies the BP-lowering effects seen with beetroot juice consumption.

The possible role of dietary nitrate supplementation *versus* conventional antihypertensive medication (or synergies with) is not studied here directly, but a non-pharmacological approach, based on a foodstuff, may be more acceptable to some patients than taking a medicine. Second, some people might not be able to tolerate antihypertensive drugs or might encounter negative effects. Beetroot juice that is high in nitrates is well tolerated and has a good safety record. Third, the combination of NR-BRJ with an antihypertensive medication may have a synergistic effect on BP.

Another important finding in the current study is that NR-BRJ may produce meaningful improvements in exercise capacity in COPD in the absence of pulmonary rehabilitation; we observed a 30 m improvement in 6MWD compared with placebo. This treatment effect exceeds the reported minimum clinically important difference for 6MWD in individuals with COPD [35], is similar to the effect of NR-BRJ seen in the context of pulmonary rehabilitation [17] and is consistent with the acute effect seen in people with COPD who require supplemental oxygen [16]. Further work is needed to see whether this improvement in exercise capacity translates into an improvement in the daily experience of physical activity. The mechanism of improvement in exercise capacity is likely to be multifactorial and may include improved skeletal muscle efficiency reducing the oxygen cost of exercise and direct effects on the circulatory system improving cardiac output by reducing ventricular loading or affecting ventilation/perfusion matching [15]. Although there was an improvement in exercise capacity, this did not translate into a statistically significant improvement in symptoms. This may be a question of sample size, but it remains to be established whether dietary nitrate supplementation can improve day-to-day symptoms in people with COPD as well as improving markers of vascular risk.

An important finding is that the fall in SBP we observed in this study was associated with improvements in a number of other accepted markers of vascular function and cardiovascular risk [36]. The improvement in endothelial function (RHI score) we observed may be due to increased NO availability following the reduction of circulating nitrate/nitrite to bioactive NO [37]. Our findings are consistent with observations in hypertensive (4 weeks; n=68) and COPD (8 weeks; n=20) patients where improvements in the percentage flow-mediated vasodilatation by 20% and 20.3%, respectively, were observed.

NR-BRJ also resulted in an improvement in the augmentation index (AIx75), a measure of arterial stiffness. Our findings corroborate the results of previous work in healthy and clinical populations [12, 30, 38]. Bahra *et al.* [38] demonstrated that a single dose (8 mmol) of oral potassium nitrate can reduce pulse wave velocity by −0.2 m·s^−1^ in healthy volunteers compared with placebo. On the other hand, dietary nitrate ingestion using beetroot juice with the same nitrate concentrations (6 mmol) in hypercholesterolaemic (n=69) and hypertensive (n=68) patients over 6 and 4 weeks lowered the augmentation index by −2.0% and −5.2%, respectively [12, 30]. While the exact underlying mechanism for this change in arterial stiffness is unclear, reports from animal mechanistic studies suggest sodium nitrite therapy reduces oxidative stress and advanced glycation end-products that are associated with arterial stiffening in aged mice [39]. Arterial compliance is suggested to be determined by both the distending pressure and the intrinsic wall properties [40]. Hence, long-term dietary nitrate-rich supplementation may alter the structural properties of blood vessels.

The increase in *F*_ENO_ observed following NR-BRJ dosing compared with placebo is in line with previous evidence from patients and healthy individuals [17, 41]. The common conclusion between these studies was that an increase in *F*_ENO_ following dietary nitrate-rich supplement is associated with an increase in plasma NO_3_^−^ level, suggesting that this could be used as a potential biomarker to monitor participants' compliance in intervention trials.

Consumption of NR-BRJ has been demonstrated to reduce platelet aggregation compared with placebo in healthy people and people with chronic conditions [12, 42]. However, we found no effect of NR-BRJ on markers of platelet activation *ex vivo*. This may be because the effects of *in vivo*-derived NO on platelet function are very short lived once platelets are removed from the body and away from sources of NO generation, because the intracellular signalling pathways involved are very rapidly reversed [43]. Elevated levels of NO within the circulation consistent with the *in vivo* effects we found could, however, reduce circulating platelet reactivity.

### Strengths and limitations

We observed a low dropout rate and high adherence to treatment which was well tolerated. Our trial used a home BP monitor to collect the BP data which is more reliable than clinic measures. Another major strength is that we assessed additional cardiovascular disease risk biomarkers including endothelial function and arterial stiffness.

We used the EndoPAT device in this study and found significant improvements in measures of endothelial function, assessed from fingertip pulse waves, with dietary nitrate supplementation. The device allows users to capture both endothelial function (RHI) and the augmentation index in a single test, and the RHI is a US Food and Drug Administration-approved test. Direct measurement of brachial artery post-occlusion dilatation may be a more sensitive technique and the physiology underpinning the two vascular responses may be different [24–27], providing a potential question for future studies. In addition, future studies should also consider the effectiveness of beetroot juice compared with conventional antihypertensive therapies as well as synergies with them.

Although the numbers were too small for subgroup comparisons in the present study, it is worth noting that people who smoke have shown a diminished increase in plasma nitrite concentration compared with non-smokers and lack any decrease in SBP in response to inorganic nitrate, in contrast to non-smokers, which may be relevant in future studies in COPD and other vascular risk populations [44]. Given that plasma nitrite values fell below the limit of accurate detectability for the assay we were only able to present total NO*_x_* in this study.

Having demonstrated efficacy in the present study, longer term multicentre studies are needed to examine the effects of beetroot juice on cardiovascular disease event rates and establish whether the improvement in exercise capacity translates into an improvement in daily physical activity and symptoms [5].

### Conclusions

Prolonged consumption of dietary nitrate in the form of beetroot juice appears to exert a sustained effect on BP and an improvement in exercise capacity and vascular function in COPD people with SBP ≥130 mmHg. These findings suggest that beetroot juice could be a candidate intervention for future health strategies to improve cardiovascular risk and prolong survival.

## Supplementary material

10.1183/13993003.02353-2022.Supp1 **Please note:** supplementary material is not edited by the Editorial Office, and is uploaded as it has been supplied by the author.
Supplementary material ERJ-02353-2022.Supplement
Study protocol ERJ-02353-2022.Protocol

## Shareable PDF

10.1183/13993003.02353-2022.Shareable1 This one-page PDF can be shared freely online.
Shareable PDF ERJ-02353-2022.Shareable
