# Supplementary material for: Oral nitrate supplementation improves cardiovascular risk markers in COPD: ON-BC, a randomised controlled trial
Source: Eur Respir J. 2024 Feb 1;63(2):2202353. doi: 10.1183/13993003.02353-2022 (PMC10831142; doi:10.1183/13993003.02353-2022)
Supplement: Supplementary file 1 [file ERJ-02353-2022.Supplement.pdf]

Oral nitrate supplementation improves cardiovascular risk markers in COPD: ON-BC a randomised controlled trial – ONLINE SUPPLEMENT

Authors: Ali M Alasmari[1,2], Abdullah S Alsulayyim[1, 3], Saeed M Alghamdi[4], Keir E J Philip[1,6], Sara C Buttery[1], Winston A S Banya[1], Michael l Polkey[1,6], Paul C Armstrong[7], M J Rickman[5], Timothy D Warner[7], J A Mitchell[5], Nicholas S Hopkinson[1].

1. National Heart and Lung Institute, Royal Brompton Campus, Imperial College London
2. College of Medical Rehabilitation Sciences, Respiratory Therapy Department, Taibah University, Madinah, Saudi Arabia
3. Faculty of Applied Medical Sciences, Respiratory Therapy Department, Jazan University, Jazan, Saudi Arabia
4. Clinical Technology Department, Umm Al-Qura University, Makkah, Saudi Arabia
5. Cardiothoracic Pharmacology, Vascular Biology, NHLI, United Kingdom
6. Respiratory Medicine, Royal Brompton and Harefield Hospitals, London, UK
7. Centre for Immunobiology, Blizard Institute, Faculty of Medicine and Dentistry, Queen Mary University of London, UK

Corresponding Author:

Professor Nicholas S Hopkinson,

National Heart and Lung Institute,

Imperial College London,

Royal Brompton Hospital Campus

Fulham Rd London SW3 6NP UK n.hopkinson@ic.ac.uk

SUPPLEMENT CONTENTS

- **Extended Methods**
- **Figure S1 The study protocol flow diagram**
- **Table S1 List Oral nitrate-based Medications**
- **Figure S2 Platelet activation test using flow cytometry.**
- **Figure S3 correlation of absolute changes in SBP and change plasma [NOx] concentration.**
- **Figure S4 A-D change in % platelet aggregation formation following dietary NO_3^- supplementation.**
- **Figure S5 change in platelet P-selectin expression following dietary NO_3^- supplementation.**
- **Table S2 Impact of dietary nitrate supplementation on clinic measures of blood pressure.**

EXTENDED METHODS

Eligibility criteria

INCLUSION CRITERIA

- Patients diagnosed with COPD based on the GOLD criteria.[1]
- Both males and females between 18 and 85 years of age.
- Able to understand and comply with protocol requirements, instructions, and protocol-stated restrictions.
- Systolic blood pressure SBP ≥ 130 mmHg via home BP monitor.

EXCLUSION CRITERIA

- Unable to provide informed consent.
- AECOPD within one month.
- Significant comorbidity limiting exercise tolerance.
- Renal impairment (estimated glomerular filtration rate (eGFR) <30 ml/min).
- Taking > three antihypertensive medications.
- Changed in BP medication in the previous month.
- Use of nitrate-based medications (Table S2).
- Use of beetroot juice drinks BRJ (Beet) Shots within one month.

Figure S1 ON-BC study protocol flow diagram

Visit 1
Review eligibility and home BP monitor (HBPM) readings obtained
Consent
Study information sheet and diary cards given
Height, weight, and body mass index (BMI) measurement.
Clinic BP
CAT and MRC scores
Spirometry
FeNO test
ECG
Oral saliva sample for microbiome profile
Endothelial function test (PAT)
6-minute walking test
Blood tests (U&E, plasma NOx, BNP, and platelet activation markers tests)

↓

Randomization
Juice delivery arranged

↙ ↘

Nitrate-rich BRJ 70mls/day

Depleted-nitrate Placebo 70mls/day

↓ ↓

6-week phone call – reinforce compliance, adverse events

↓ ↓

11-week phone reminder for home 4-day BP monitoring

↓ ↓

Visit 2
Collect diary cards for juice compliance
Collect HBPM post-intervention readings
CAT and MRC scores
FeNO test
ECG
Weight, and body mass index (BMI) measurement.
Clinic BP
Oral saliva sample for microbiome profile
Endothelial function test (PAT)
6-minute walk test (final test)
Blood tests (U&E, plasma NOx, BNP, and platelet activation markers tests)

End of study

Table S1 List Oral nitrate-based Medications

Medication class	Brand name
Glyceryl Trinitrate	Nitro-Dur®, Minitran®, Transiderm-Nitro®, Deponit®, and Percutol®
Isosorbide Mononitrate	Imdur®, Ismo Retard®, Isotard®, Monomil®, XL Chemydur®, 60XLModisal®, XLMonomax®, XLIsib®, 60XLMonosorb®, XL60 Zemon®, Isodur®, and Elantan® LA
Nicorandil	Ikorel®

ENDOTHELIAL FUNCTION – EXTENDED METHODS

The EndoPAT is an FDA-cleared non-invasive device, user-independent, and intended for use as a diagnostic aid in the detection of endothelial dysfunction[2]. It uses computerised analysis of endothelial function via peripheral arterial tone (PAT) in response to induced reactive hyperaemia.

PAT is measured via the fingertip by recording pulse wave amplitude signals and reported as reactive hyperaemia index (RHI) score with arbitrary units. Pulse amplitude response to hyperaemia is automatically calculated from the hyperaemia in the finger of the experimental arm as a ratio of the post-deflation average pulse amplitude to the baseline average pulse amplitude. This result was divided by the corresponding ratio from the contralateral control hand to obtain the RHI score.

In addition, the EndoPat software also provides an augmentation index (AIx75), a surrogate measure of arterial stiffness, by estimating the timing and magnitude of the pulse wave reflection. It is calculated from the average baseline resting PWA data over 3.5 min before cuff occlusion following this formula: (P1–P2/ P1) × 100, where P1 = systolic peak pressure and P2 = the reflected peak pressure. Then, the values were adjusted to a standard heart rate of 75 beats/min (AIx75).

Participants, upon arrival to do the test, rested for 10 min in a quiet, temperature-controlled (21–24°C) room with dimmed lights and were asked to remain as still as possible and silent during the entire measurement period. Each recorded test consisted of 5 minutes of baseline measurement, 5 minutes of occlusion measurement, and 5 minutes post-occlusion measurement (hyperaemic period). Occlusion of the brachial artery was performed on the

non-dominant upper arm. The occlusion pressure was at least 60 mmHg above the SBP (minimally 200 mmHg, and maximally 300 mmHg). A computerised automated algorithm was generated to automatically calculate the RHI value by dividing post-PWA by the pre-occlusion value of PWA of the same arm, normalised to the control arm, and then multiplied by the baseline correction factor. Absolute endothelial dysfunction was defined as an RHI score of less than 1.67. The measurements were carried out according to the published manufacturer's protocol.[2]

BIOMARKERS

Plasma samples were obtained through a 21-gauge butterfly needle inserted into an antecubital vein in a labelled (4 ml) EDTA vacutainer blood tube.

Plasma NOx concentrations

Plasma NOx concentrations were assessed by the Griess method characterised by high reproducibility and minimised interferences by plasma constituents [3]. We utilised the nitrate/nitrite colorimetric assay kit from (Cayman Chemical, Ann Arbour, MI, USA) for high reproducibility and minimised interferences by plasma constituents. All samples in EDTA were defrosted and subjected to ultrafiltration using a 10–kDa filter from Merk Millipore Ltd. (Tullagreen, Ireland). The total nitrate/nitrite concentrations were determined by two-step processes: (a) the conversion of nitrate to nitrite by NADH-dependent nitrate reductase enzyme, and (b) the addition of Griess reagents, which convert nitrite to a deep purple azo product. The absorbance at 540 nm due to the azochromophore was measured by a microplate reader (TECAN Infinite M200PRO, Grödig, Austria). All absorbance was then plotted on the standard curves. Nitrate was calculated by subtracting the nitrite from [NO_x].

Analysis of platelet activation biomarkers

We tested the following biomarkers: platelet aggregator % and P-selectin expression % on ex vivo whole blood 96-well plate measured via flow cytometry.[4] Most of this test process and analysis were done at Professor Tim Warner, vascular research lab, Queen Mary University, London, UK. This method incorporates a range of agonists at different concentrations, which will individually test multiple pathways of platelet activation (Figure S1).

Figure S2 Platelet activation test using flowcytometry approach.

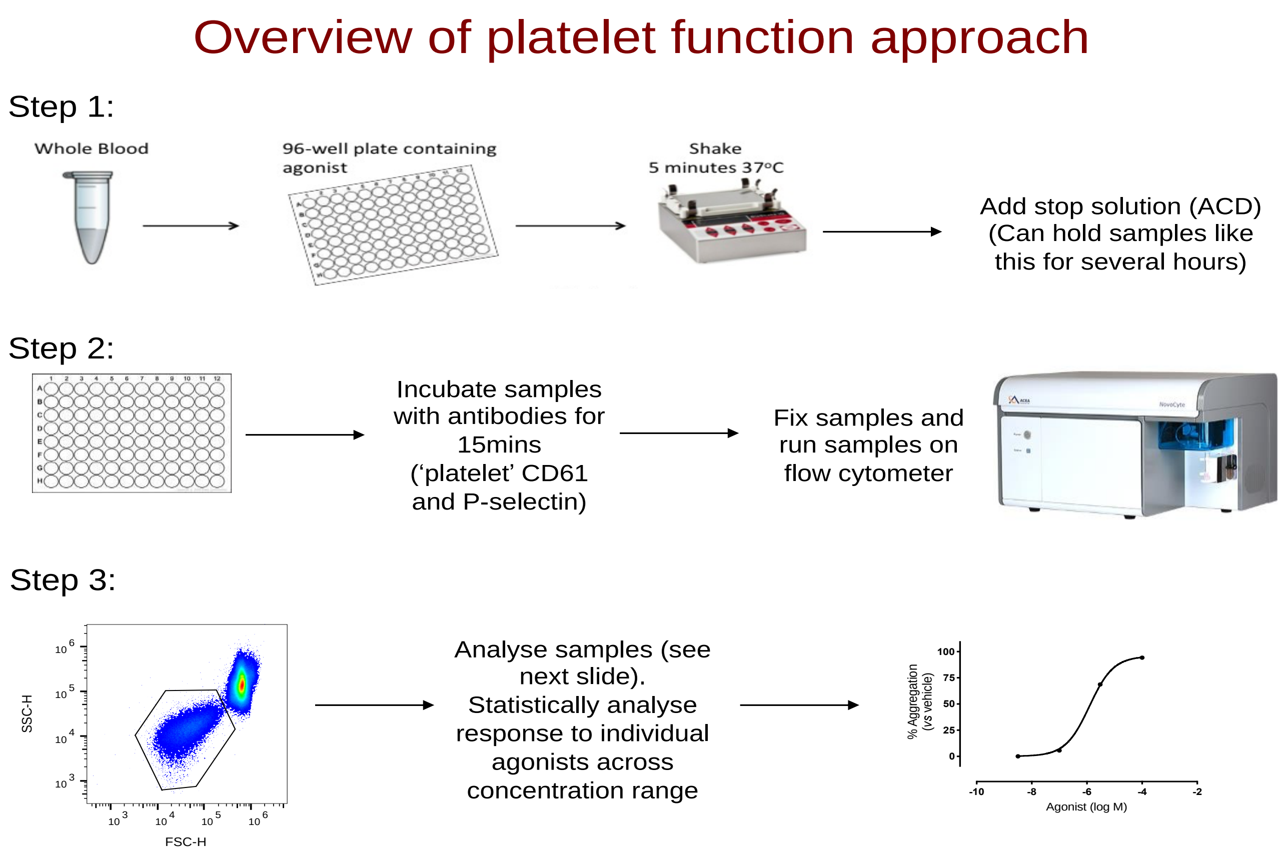

Participants' blood collected into a 3.2% sodium citrate blood collection container through a 21-gauge butterfly needle inserted into an antecubital vein. Following venepuncture, blood was pipetted (40µl) into agonist-containing wells to initiate aggregations. Aggregation was

stimulated by arachidonic acid (0.03, 0.1, 0.3, 0.6µM), collagen (0.1, 0.3, 1, 3µg/ml), thrombin PAR1-receptor activating peptide (TRAP; 0.1, 0.3, 1, 3µM) and thromboxane A_2 mimetic U46619 (0.1, 0.3, 1, 3µM). Then, the plate is placed on a plate shaker (BioShake iQ, Q instruments) for 5 min at 1000 rpm and 37℃. After 5 min, 160µl of pre-prepared acid citrate dextrose (ACD) solution was added to each well. This solution acts as a 'stop-solution' to preserve aggregation and prevent any further activation. Samples in this state were stable for up to six days before further processing. Aggregation was determined using flow cytometry. 10µl of each experimental well was transferred into a separate well containing 10µl anti-CD61 (1:25; clone VI-PL2, Biolegend) monoclonal antibody conjugated to allophycocyanin (APC) in PBS to label and identify the platelet population. Samples were stained at room temperature for 30 min before fixing with 180µl of a BSA/dextrose saline solution containing 0.1% formalin (Sigma-Aldrich). Platelets were identified by forward and side scatter and CD61 positivity using a Novocyte 3000 flow cytometer (ACEA), and totals were counted in 10µl of each experimental well sample.

P-selectin expression %

Whole blood collected from individuals was placed in prepared wells containing EDTA and either vehicle (PBS), ADP (0.5µM or 40µM), or TRAP-6 (40µM). Then, the plate was placed on a plate shaker (BioShake iQ, Q instruments) for 5 min at 1000 rpm and 37℃. After 5 min, 160µL of pre-prepared acid citrate dextrose (ACD) solution was added to each well. Samples in this state were stable for up to six days before further processing.

As for aggregation samples, 10µl of each experimental well was transferred into a separate well containing 10µl anti-CD61-APC (1:25; clone VI-PL2, Biolegend) and anti-CD62P-PE (1:25; clone AK-4, Biolegend) or isotype control (1:25, clone MOP-21, Biolegend) for the identification of platelets and quantification of P-selectin expression. Samples were analysed

using a Novocyte 3000 flow cytometer (ACEA), with expression levels calculated from 10,000 acquired platelet events. The results were expressed as the percentage of platelets positive for P-selectin.

Figure S3 Changes in HSBP and change plasma NOx concentration

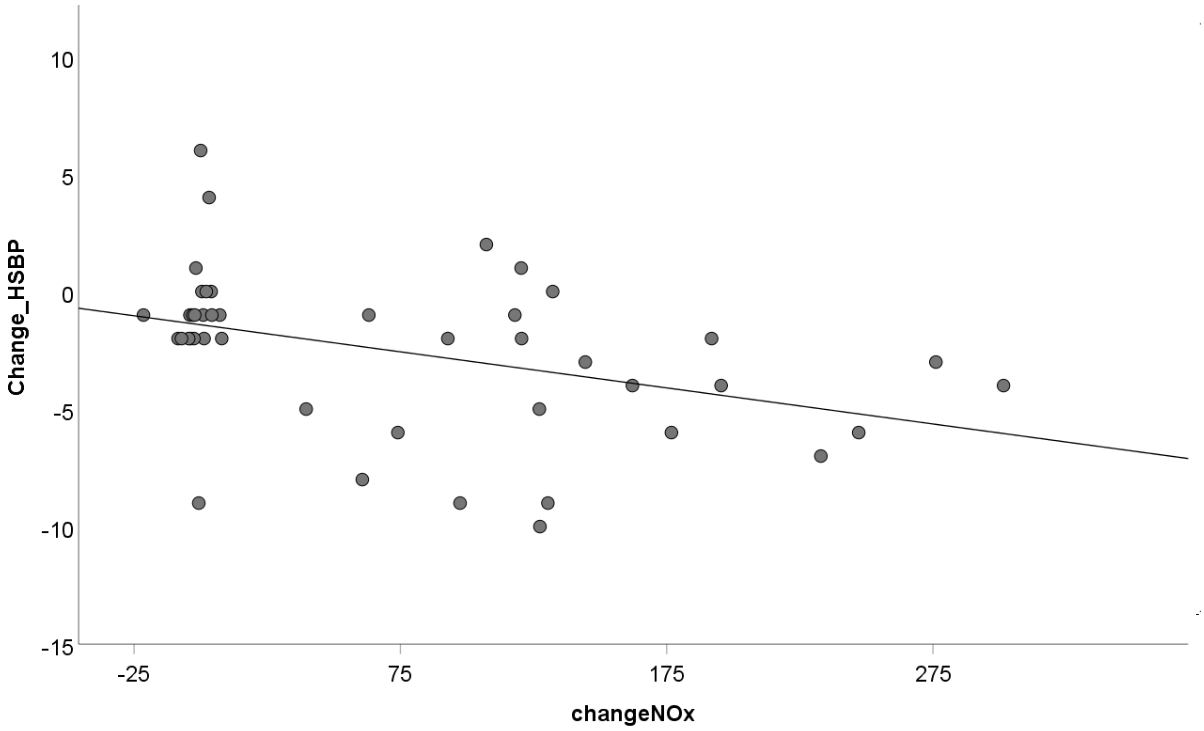

Association between change in NOx (µM) levels and change in home monitored systolic blood pressure (HSBP) R -0.402 (p=0.007). Values for six minute walk distance (6MWD) R +0.487 (p=0.001), Reactive hyperemic index (RHI) R +0.293 (p=0.067) and augmentation index (AIx75) R -0.255 (p=0.113)

Figure S4 A-D change in % platelet aggregation formation following dietary NO_3^- supplementation.

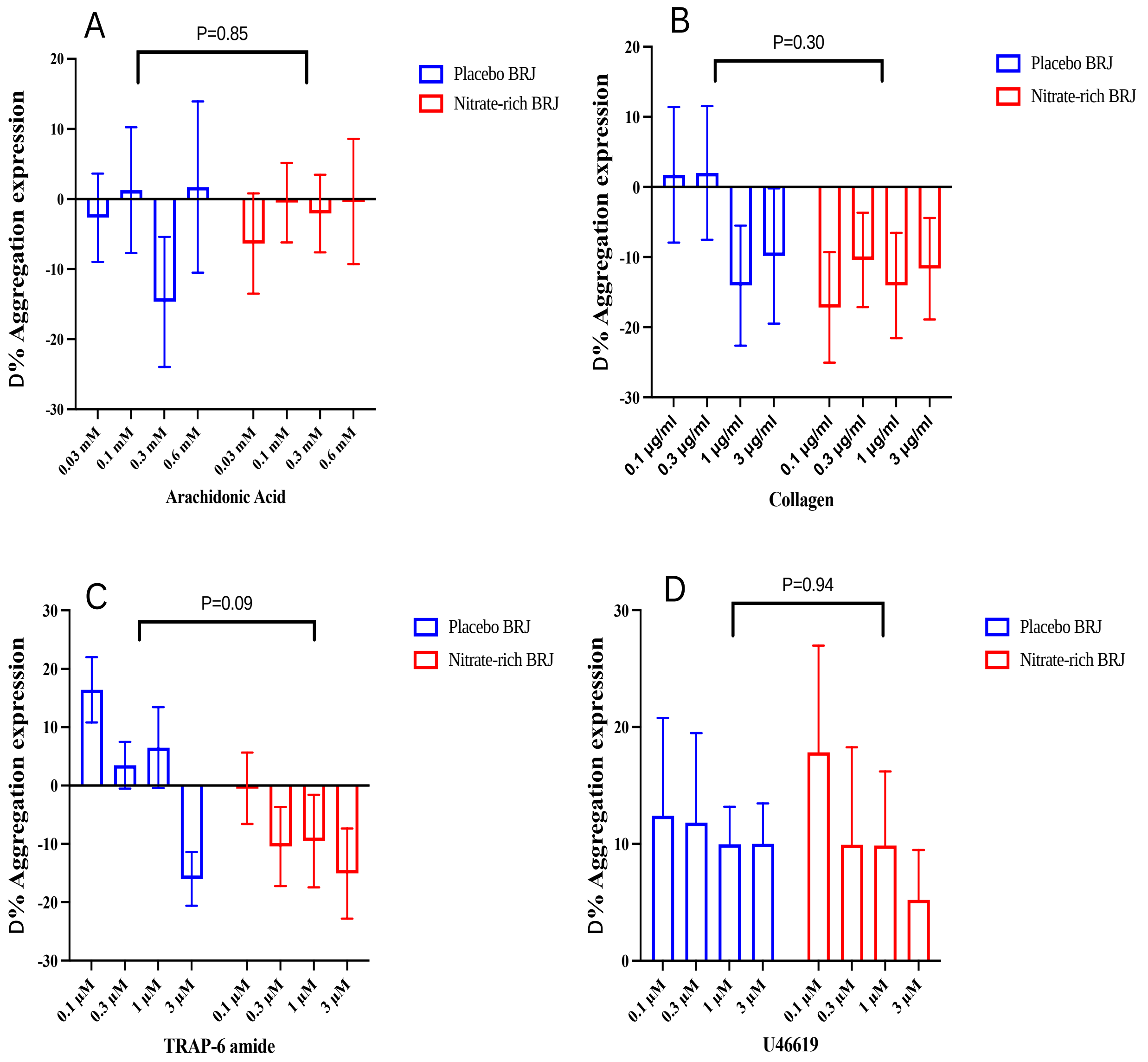

Data shown are placebo BRJ (n = 17) vs 70 mL nitrate-rich BRJ (n = 19) expressed mean ± SEM. Ex vivo stimulation of platelets in small volumes of whole blood in 96-well plates measured with flow cytometry in response to the following agonist concentrations: arachidonic acid (0.03–0.6 µM), collagen (0.1- 3 µg/ml), TRAP-6 amide (0.1- 3 µM), and thromboxane A2 mimetic U46619 (0.1- 3 µM). P-value for the comparison between groups using two-way ANOVA with Bonferroni post-tests.

Figure S5 change in platelet P-selectin expression following dietary NO_3^- supplementation.

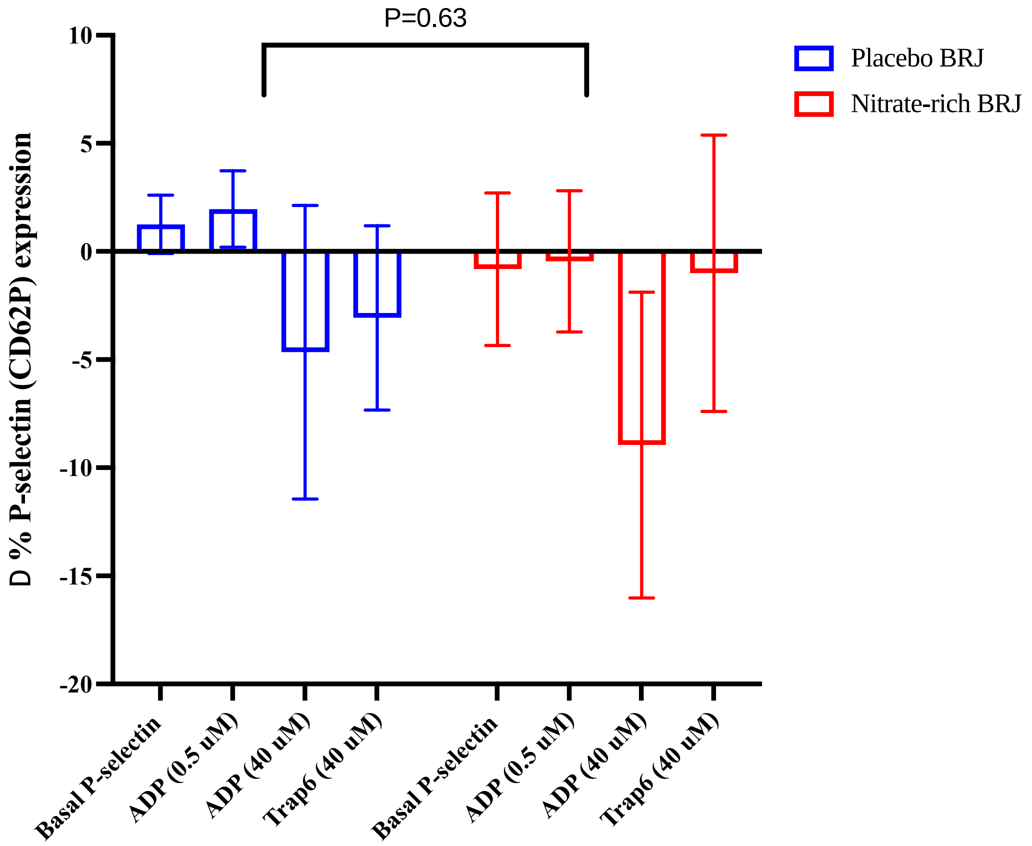

Data shown are placebo BRJ (n = 17) vs 70 mL nitrate-rich BRJ (n = 19) expressed mean ± SEM. Ex vivo stimulation of P-selectin in response to following agonists vehicle, ADP 0.5µM, ADP 40µM TRAP-6 40µM. P-value for the comparison between groups using two-way ANOVA with Bonferroni post hoc tests.

Table S2 Impact of dietary nitrate supplementation on clinic measures of blood pressure.

Variable	Pl-BRJ (n=20)			NR-BRJ (n=24)			**Treatment Effect (95% ci)**	p
	Pre	Post	Difference	Pre	Post	Difference		
CSBP mmHg	140.7 ± 17.9	138.5 ± 16.7	-2.2 ± 13.3	137.4 ± 12.9	130.2 ± 12.7	-7.2 ± 10.4	-5.0 (-12.3 to 2.2)	0.17
CDBP mmHg	74.7 ± 10.3	77.5 ± 10.0	3 (-7, 13.6)	79.3 ± 9.1	75.9 ± 10.3	-2 (-8.1, 1.0)	-6 (-13 to -1)	0.025

CSBP – clinic visit systolic blood pressure

CDBP – clinic visit diastolic blood pressure

Numeric data were presented as mean (± SD) or as median (IQR) depending on the distribution of the data. The treatment effect was estimated by subtracting the baseline values from the 12 week values, then using a 2-sample independent t test or the Wilcoxon rank-sum test to compare the groups. Where the t test was used the treatment effect was reported as the difference between the 2 means with its 95% confidence interval and where the Wilcoxon rank-sum test was used the treatment effect was estimated with the Hodges-Lehman estimate. The Hodges-Lehman process entails estimating the average difference in outcomes (x-y) for every possible n(n+1)/2 pair and then deriving the overall median of all averages (the Hodges-Lehmann estimator). A distribution-free confidence interval is estimated using large-sample approximation.

REFERENCES

1. Vestbo J, Hurd SS, Agusti AG, et al. Global strategy for the diagnosis, management, and prevention of chronic obstructive pulmonary disease: GOLD executive summary. *Am J Respir Crit Care Med.* 2013;187(4):347-365.
2. Allan R, Delaney C, Miller M, Spark J. A comparison of flow-mediated dilatation and peripheral artery tonometry for measurement of endothelial function in healthy individuals and patients with peripheral arterial disease. *European Journal of Vascular and Endovascular Surgery.* 2013;45(3):263-269.
3. Giustarini D, Rossi R, Milzani A, Dalle-Donne I. Nitrite and nitrate measurement by Griess reagent in human plasma: evaluation of interferences and standardization. *Methods in enzymology.* 2008;440:361-380.
4. Armstrong PCJ, Kirkby NS, Chan MV, et al. Novel whole blood assay for phenotyping platelet reactivity in mice identifies ICAM-1 as a mediator of platelet-monocyte interaction. *Blood, The Journal of the American Society of Hematology.* 2015;126(10):e11-e18.
