# Supplementary material for: Oral nitrate supplementation improves cardiovascular risk markers in COPD: ON-BC, a randomised controlled trial
Source: Eur Respir J. 2024 Feb 1;63(2):2202353. doi: 10.1183/13993003.02353-2022 (PMC10831142; doi:10.1183/13993003.02353-2022)
Supplement: Supplementary file 2 [file ERJ-02353-2022.Protocol.pdf]

The ON-BC study

Oral Nitrate supplementation and Blood pressure in COPD – a randomised clinical trial

Version 2 24th June 2020

MAIN SPONSOR: Imperial College London

FUNDERS: The Saudi Arabian Cultural Bureau

STUDY COORDINATION CENTRE: NHLI, Imperial College, Royal Brompton Hospital Campus

IRAS Project ID:	271589
REC reference:	19/LO/1660
IC JRO reference number:	19IC5484

Protocol authorised by:

Name & Role

Dr Nicholas Hopkinson
Principal Investigator

Study Management Group

Chief Investigator: Dr Nicholas Hopkinson

Co-investigators: Mr Ali Alasmari
Professor Mike Polkey

Statistician: Winston Banya

Study Coordination Centre

For general queries, supply of trial documentation, and collection of data, please contact:
Study Coordinator: Ali Alasmari
Address: Royal Brompton Hospital, Fulham Rd, London. SW3 6NP
Tel: 020 73518029 E-mail: a.alasmari18@imperial.ac.uk Fax: 020 73518939

Clinical Queries

Clinical queries should be directed to Ali Alasmari who will direct the query to the appropriate person.

Sponsor

Imperial College London is the main research Sponsor for this study.
For further information regarding the sponsorship conditions, please contact the Head of Regulatory Compliance at:

Joint Research Compliance Office
Imperial College London and Imperial College Healthcare NHS Trust
Room 215, Level 2, Medical School Building
Norfolk Place
London, W2 1PG
Tel: **0207 594 9480**
http://www3.imperial.ac.uk/clinicalresearchgovernanceoffice

Funder

The study is funded by the Saudi Arabian Cultural Bureau

This protocol describes the ON-BC study and provides information about procedures for entering participants. The protocol should not be used as a guide for the treatment of other participants; every care was taken in its drafting, but corrections or amendments may be necessary. These will be circulated to investigators in the study, but centres entering participants for the first time are advised to contact the trials centre to confirm they have the most recent version.
Problems relating to this trial should be referred, in the first instance, to the study coordination centre.
This trial will adhere to the principles outlined in the Medicines for Human Use (Clinical Trials) Regulations 2004 (SI 2004/1031), amended regulations (SI 2006/1928) and the International Conference on Harmonisation Good Clinical Practice (ICH GCP) guidelines. It will be conducted in compliance with the protocol, the Data Protection Act and other regulatory requirements as appropriate.

Table of Contents Page No

**1.**	**INTRODUCTION**	**9**
1.1	BACKGROUND	9
**2.**	**STUDY OBJECTIVES**	**13**
**3.**	**STUDY DESIGN**	**13**
3.1	STUDY OUTCOME MEASURES	14
**4.**	**PARTICIPANT ENTRY**	**14**
4.1	PRE-RANDOMISATION EVALUATIONS	14
4.2	INCLUSION CRITERIA	14
4.3	EXCLUSION CRITERIA	14
4.4	WITHDRAWAL CRITERIA	15
**5.**	**RANDOMISATION AND ENROLMENT PROCEDURE**	**15**
5.1	RANDOMISATION OR REGISTRATION PRACTICALITIES	15
5.2	UNBLINDING	15
**6.**	**TREATMENTS**	**15**
6.1	TREATMENT ARMS	15
6.2	INTERACTION WITH OTHER DRUGS	15
6.3	DISPENSING AND ACCOUNTABILITY	15
**7.**	**ADVERSE EVENTS**	**15**
7.1	DEFINITIONS	15
**8.**	**ASSESSMENT AND FOLLOW-UP**	**17**
8.1	DETAILS OF ASSESSMENTS	20
8.2	LOSS TO FOLLOW-UP	22
8.3	TRIAL CLOSURE	23
**9.**	**STATISTICS AND DATA ANALYSIS**	**23**
**10.**	**MONITORING**	**23**
10.1	RISK ASSESSMENT	23
10.2	MONITORING AT STUDY COORDINATION CENTRE	23
**11.**	**REGULATORY ISSUES**	**23**
11.1	ETHICS APPROVAL	23
11.3	CONSENT	24
11.4	CONFIDENTIALITY	24
11.5	INDEMNITY	24
11.6	SPONSOR	24
11.7	FUNDING	24
11.8	AUDITS AND INSPECTIONS	24

12. TRIAL MANAGEMENT 24

13. PUBLICATION POLICY 24

14. REFERENCES 25

APPENDIX 1. SUMMARY OF INVESTIGATIONS, TREATMENT AND ASSESSMENTS 28

GLOSSARY OF ABBREVIATIONS

ADMA	Asymmetric dimethylarginine
ADP	Adenosine diphosphate
AECOPD	Acute exacerbation COPD
BMI	Body mass index
BNP	Brain Natriuretic Peptide
BP	Blood pressure
BRJ	Beetroot juice
CAT	COPD assessment test
COPD	Chronic obstructive pulmonary disease
CVD	Cardiovascular disease
DBP	Diastolic blood pressure
eNOS	Endothelial nitric oxide synthase
FeNO	Fractional exhaled nitric oxide
FEV_1	Forced expiratory volume in one second
FMD	Flow Mediated Dilatation
GFR	Glomerular filtration rate
GOLD	Global Initiative for Chronic Obstructive Lung Disease
HRQoL	Health related quality of life (HRQoL)
IHD	Ischaemic heart disease
IP	Inorganic phosphate
L-NMMA	L-NG-monomethylarginine
MAP	Mean arterial pressure
MRS	Magnetic resonance spectroscopy
NIRS	Near-infrared spectroscopy
NO	Nitric oxide
NOS	Nitric oxide synthase
PA	Physical activity
PAD	Peripheral arterial disease
PCr	Phosphocreatine
PFT’s	Pulmonary function tests
PHT	Pulmonary hypertension
PL	Placebo
PR	Pulmonary rehabilitation
6MWD	six minutes-walk test distance
SBP	Systolic blood pressure diastolic
XO	Xanthine oxidase

KEYWORDS

COPD, nitrate, cardiovascular disease, exercise

STUDY SUMMARY

TITLE Oral Nitrate supplementation and blood pressure in COPD – a randomised clinical trial (The ON-BC study).

DESIGN A double blind, placebo-controlled, parallel group study of the effect of nitrate supplementation on markers of cardiovascular risk in patients with stable COPD

AIMS To establish whether, in people with stable COPD, dietary nitrate supplementation, in the form of beetroot juice administered for 3 months will: (1) reduce BP, (2) improve exercise capacity (3) improve endothelial function (4) inhibit platelet aggregation

OUTCOME MEASURES

Primary endpoint Change in home monitored BP.

Secondary endpoints

i) Exercise capacity (6 minute walk test distance)
ii) Health related quality of life (HRQoL)

Exploratory endpoints

i) Endothelial function (Endopat score)
ii) Blood Brain Natriuretic Peptide levels (BNP)
iii) Markers of platelet activation (platelet-monocyte aggregates)
iv) Plasma concentration of arginine/asymmetric dimethylarginine (ADMA) levels)
v) Fractional exhaled NO (FeNO)
vi) Blood nitrate and nitrite levels
vii) Oral microbiome sampling

POPULATION 72 adult patients with GOLD I-IV COPD

ELIGIBILITY *Inclusion*

- adult patients (>21 yrs) with stable COPD GOLD I-IV
- established on stable pharmacotherapy for COPD
- Systolic blood pressure >130 mmHg

Exclusion

- Unable to provide informed consent.
- AECOPD in the preceding month
- Significant comorbidity limiting exercise tolerance
- Significant comorbidity limiting life expectancy
- Significant renal impairment (estimated glomerular filtration rate (eGFR) <30 $ml.min^{1}$)
- Use of >3 blood pressure lowering medications
- Change in medication in the previous month
- Oral nitrate medication
- Current (in the last month) use of Beet Shots.

TREATMENT Active: 70mls Beet It Stamina shot from James White Ltd (6.5mmol Nitrate)
Placebo: 70mls matched placebo shot with nitrate removed.

DURATION 3 months

REFERENCE DIAGRAM

Visit 1
Review eligibility
Consent
ECG CAT score Spirometry FeNO test
Height, weight, and body mass index (BMI) measurement.
Blood tests (U&E, BNP, platelet function, nitrate levels)
Oral samples for microbiome
6 minute walk test
Home BP monitor (4 days) commences

↓

Visit 2
Clinic BP
Endothelial function

↓

Randomisation Stratified by site and prior use of BP lowering medication

↙ ↘

BRJ 70mls/day | **Placebo 70mls/day**

↓

6 week phone call – reinforce drinking

↓

11 week phone reminder for home 4 day BP monitoring

↓

Visit 3
Collect empty bottles for compliance count
Collect home BP monitor (4 days)
ECG CAT score FeNO test
Weight, and body mass index (BMI) measurement.
Clinic BP
Endothelial function
Blood tests (U&E, BNP, platelet function, nitrate levels)
Oral samples for microbiome
6 minute walk test (final test)

End of study

Lay Summary

Background: Dietary nitrate supplementation, in the form of beetroot juice, has a number of potentially advantageous effects in COPD. These include improving the response to pulmonary rehabilitation programme, making muscle contraction more efficient so it uses less oxygen, and improving how far people with low oxygen levels because of their lung disease can walk. Although COPD is a lung disease, people with the condition are at a higher risk of heart disease and stroke. There is also some evidence that beetroot juice can reduce blood pressure, but studies so far have been short term. A nutritional treatment that could produce a lasting reduction in blood pressure would be appealing, especially if it also improves people's ability to exercise.

Aims: The purpose of this study is to investigate the prolonged treatment effects of daily beetroot juice on blood pressure in people with COPD. We will also look at how far people can walk. We will make measurements of how well blood vessels function and take blood samples to look at the mechanisms involved including how "sticky" platelets are. These are the cells in the blood that cause it to clot.

Study Design: 72 patients with COPD will be enrolled in the study. Half will drink a 70ml beetroot juice "shot" each morning for three months. This contains 6.5mmol nitrate, the active ingredient. The other half will take identical juice drink which has had the nitrate removed. Which group participants are in will be decided at random by a computer. The main outcome will be blood pressure measured by participants at home for 4 days at the beginning and end of the study. In addition we will measure how far people can walk, how well blood vessels work using a device that measures blood flow, and blood tests looking at nitrate levels and platelet function. We will also collect mouth swabs to look at bacteria in the mouth to see if that changes with treatment. The study is funded by The Saudi Cultural Centre.

Expected outcome: If the study is positive this will help in the development of beetroot juice as a therapy for people with COPD and other long term conditions.

1. INTRODUCTION

1.1 BACKGROUND

COPD is a common condition responsible for considerable morbidity and mortality. There are 1.3 million people with a diagnosis of COPD in the UK and existing treatments are relatively ineffective. The presence of multiple medical problems in COPD has become the norm rather than the exception, and 50% of patients have three or more other long term conditions [1]. We have shown that a novel approach, dietary nitrate supplementation in the form of beetroot juice, has a number of potentially advantageous effects in COPD. These include enhancing the effect of pulmonary rehabilitation on exercise capacity (the ON-EPIC trial), reducing the oxygen cost of exercise in people with less severe COPD [2]and improving exercise capacity in hypoxic COPD patients (EDEN-OX trial).

At least 40% of COPD patients have concomitant cardiovascular disease[1]. Cardiovascular disease is the cause of death in about one third of individuals with COPD [3,4]. Although smoking and social deprivation are common risk factors for both conditions, COPD does appear to be an independent risk factor for cardiovascular disease [5]. Outcomes in patients with COPD are worse after acute coronary episodes than conventional scoring systems (the GRACE score) would anticipate, again suggesting that COPD should be treated as a risk marker, like diabetes.[6] Dietary nitrate supplementation has been shown to reduce blood pressure both in normotensive[7] and hypertensive[8] subjects, though studies to date have been of relatively short duration – the longest for 5 weeks (reviewed here [9,10]). There are also data linking NO with the development of metabolic syndrome - a clinical and biochemical expression of insulin resistance, representing a clustering of central obesity, hypertension, hyperglycemia, and dyslipidemia.[11,12]

Beetroot juice (BRJ) drinks are commercially available, marketed for their potential health benefits and widely used by sports people. The Beet It Stamina shot from James White Ltd is 70mls and contains 6.5mmol nitrate. A matched placebo beetroot beverage is available with identical appearance and taste, but which has been passed through an ion exchange column to remove the active nitrate component.

INTRODUCTION TO NITRATE METABOLISM

Nitric oxide (NO) is an important physiological mediator in the body. NO is endogenously produced via the action of the NO synthase (NOS) family of enzymes acting on the amino acid L-arginine, including endothelial NOS (eNOS), neuronal NOS (nNOS) and inducible NOS (iNOS). These enzymes catalyse the conversion of arginine to NO and L-citrulline in a reaction dependent on the presence of molecular oxygen and several cofactors[13]. Endothelial NOS (eNOS) is itself activated by shear stress on the endothelial membrane, leading to NO production and vasodilatation[14,15].

In addition to the action of the NOS enzyme family, NO is produced via the reduction of exogenous dietary nitrate (NO_3^-) in an oxygen independent manner (Figure 1). Nitrate can be found at high levels in leafy greens and beetroot, with vegetables providing 60-80% of our daily dietary intake[16]. Ingested inorganic nitrate passes readily into the circulation, with the majority being renally excreted[17,18]. Approximately 25% of ingested nitrate is actively taken up by the salivary glands via the enterosalivary circulation[18], where concentrations may be over 10-fold greater than those measured in the plasma. It is then excreted in saliva and reduced from inert nitrate to bioactive nitrite (NO_2^-) via nitrate reductases produced by oral commensal facultative anaerobic bacteria[19,20]. Salivary nitrite may be absorbed as saliva is

swallowed and enter the circulation directly as nitrite[19], or be reduced to nitrogen oxides such as NO in the acidic environment of the stomach[21-23] and then absorbed. Thus the supplementation of the diet with nitrate provides a means of increasing circulating plasma nitrite levels which are an indicator of bioavailable NO. The importance of commensal bacteria in facilitating the metabolism of nitrate can be demonstrated by the prevention of the normal rise in plasma nitrite levels after an oral nitrate bolus following the administration of an antibacterial mouthwash[24].

The reduction of plasma nitrite to NO is a process enhanced in hypoxic and acidic conditions as may occur in tissues during exercise[25,26]. This is an important process as under such conditions hypoxia may be limiting NO generation via the oxygen-dependent NOS pathway[27].

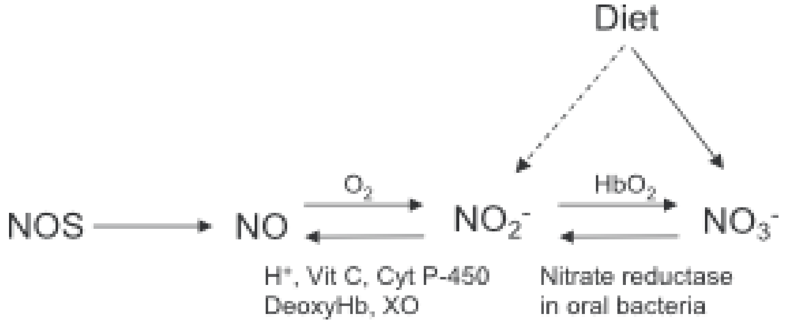

Figure 1. Nitrate-nitrite-NO pathway in humans. Plasma nitrate originates from the oxidation of endogenously produced NO and dietary sources. Nitrite reduction to NO is enhanced in acidic (H^+), hypoxic and reducing (e.g. vitamin C) conditions. Other proteins and enzymes may promote this step including Cytochrome P-450 (Cyt P-450), Xanthine Oxidase (XO) and Deoxyhaemoglobin (DeoxyHb). Taken from Lundberg et al.[19]

Thus there are two common nitrate-nitrite-NO pathways, via the NOS family of enzymes and via reduction of dietary nitrate. In fact where endogenous NO production is limited via the knock-out of the eNOS gene, the supplementation of nitrite[28] or nitrate[29] enhances plasma nitrite and nitrate levels towards those seen in normal subjects. This would suggest there is an important interplay between the endogenous and exogenous sources of NO production. Although health concerns have been raised regarding supplementation with pure sodium nitrate or nitrite due to increased generation of *N*-nitrosamines with increased risk of gastric cancer, the generation of nitric oxides from nitrite is thought to be an important physiological pathway in humans. In fact it has been proposed that nitrite is an important source of reactive nitrogen intermediates including NO that possess important antimicrobial activity, gastric protective effects and control of vascular tone particularly in ischaemic tissues[22]. In addition nitrate-rich whole vegetables or juices also contain antioxidants and polyphenols that may reduce the formation of harmful nitrogenous compounds[30,31]. Thus the general consensus is that nitrate supplementation from vegetable sources is likely to be an important component of a healthy diet.

Abnormalities in NO synthesis occur in ageing and may be linked to poor exercise tolerance seen in old age. Older subjects show reduced plasma nitrite production in response to exercise than younger individuals [32]. As nitrite is the main oxidation product of NO this is reflective of reduced eNOS activity during exertion, and as such can be abolished by the systemic administration of the NOS inhibitor L-NMMA (L-NG-monomethylarginine). This reduced NOS activity is accompanied by impaired endothelial vasodilator function in response to exercise in older persons as assessed by brachial artery flow mediated dilation studies[32].

Impact of nitrate supplementation on exercise in COPD.

NO is key regulator of skeletal muscle blood flow, contractility, glucose and calcium haemostasis and mitochondrial function [33]. The landmark study of nitrate supplementation by Larsen *et al* (2007)[34] found that, in healthy individuals, acute nitrate supplementation reduced the oxygen cost of submaximal exercise. A finding confirmed in subsequent healthy subject trials [35].

Patients with chronic obstructive pulmonary disease (COPD) have symptoms of exercise limitation that affect their activities of daily living. Exercise training through pulmonary rehabilitation (PR) is a well-established and effective therapy to ameliorate this[36]. Various research groups have investigated the role of dietary nitrate supplementation in COPD. We found that dietary nitrate supplementation reduced the oxygen cost of sub-maximal exercise assessed via cycle ergometry albeit without increasing exercise capacity in patients with COPD[2]. Berry *et al* also assessed the effect of dietary nitrate supplementation on sub-maximal exercise via cycle ergometry and found an increase in exercise capacity [37]. Kerley *et al* also found an increase in exercise capacity as assessed by an incremental shuttle walk test [38]. In another study, a week of BRJ 140mls BD lowered DBP but did not improve 6MWD in COPD, not using oxygen, mean FEV_1 44.7±15.1.[39]

In the ON-EPIC trial (presented ERS 2018) we randomised patients participating in pulmonary rehabilitation to take 140mls of BRJ or matched placebo before each exercise session. At the end of the program the improvement in exercise capacity in the active group, assessed using the incremental shuttle walk test (ISWT), was *double* that seen in the placebo arm: nitrate group +60.3 m (-110.0, 180.0) vs. placebo +30.0 m (-70.0, 290.0), p = 0.011.

Furthermore, in a cross over study in hypoxic patients (EDEN-OX) (presented ERS 2018), we found that 140mls beetroot supplementation increased endurance shuttle walk time substantially compared to placebo, with an estimated treatment effect of 62sec (95%CI 33, 106) p<0.0001.

There is therefore a compelling evidence base to support pursuing the hypothesis that oral nitrate supplementation with BRJ may improve exercise performance in COPD.

Beetroot juice – vascular impact in COPD

Several studies support a short term effect of nitrate supplementation in the form of beetroot juice on blood pressure in COPD. We have demonstrated that nitrate supplementation lowers the oxygen cost of exercise in COPD[40]. In that single dosing study diastolic blood pressure (DBP) fell by 7 ± 8 mmHg. Another study in COPD patients, using 3 days dosing with twice daily 4.8 mmol nitrate 70 mL beetroot juice, found a 10mmHg reduction systolic blood pressure (SBP) [41]. Kerley et al. used acute doses of 12.9 mmol and SBP, DBP and mean arterial pressure (MAP) decreased by −12 ± 19; −1.6 ± 16; −5 ± 4 mmHg respectively[42]. Berry et al., used a single dose of 7.58 mmol and found SDP was 8mmHg lower and DBP 3mmHg lower.[37] A week of Beetroot 140mls BD lowered DBP by a mean 4.6mmHg[39]. One study used 6.77 mmol twice a day for 2.5 days and found no difference in blood pressure.[43] COPD also involves damage to the pulmonary vascular bed which can progress to pulmonary hypertension [44]. There is intriguing data, in idiopathic pulmonary hypertension, to show a link between low circulating nitrate levels and both the extent of pulmonary hypertension and its progression and mortality.[45]

An outstanding question is whether dietary nitrate supplementation will cause a sustained fall in blood pressure associated with a reduction/reversal in end organ damage (arterial stiffness).

The oral microbiome and nitrate metabolism

The community of microorganisms that inhabit the human body are referred to as the human microbiome. It consists mainly of bacteria, fungi and viruses and has been associated with both health and disease. Between individuals there is a common catalogue of microorganisms, but which any one person has and the relative abundances of each of these is unique to them [46,47]. In processes mediated by bacteria, such as nitrate reduction, this uniqueness is a potential source of variation as different microbial communities are not always functionally identical.[48] Previous investigators have noted a change in the composition of the oral microbiome in response to nitrate supplementation in a modest cohort, with an increase in the genera *Neisseria* and *Rothia*[49]. Smoking has been demonstrated to have an impact on the oral microbiome with selective depletion of certain organisms including *Neisseria*.[50] There is little data on the composition of the COPD oral microbiome, as usually the lung microbiome is studied in these individuals, so which organisms are responsible for the conversion of nitrate and how they vary between subjects will be determined here. If the oral microbiome is indeed a source of variation in response to nitrate supplementation then it might be possible to identify a nitrate reducing strain that could be used as a probiotic to normalise the nitrate-nitrite-NO functional pathway prior to supplementation with nitrate.

Factors potentially influencing the oral microbiota in COPD and thus nitrate/nitrite metabolism include smoking history, poor dentition, inhaled medication, gastric acid suppressing medication and gastro-oesophageal reflux. Advances in understanding of the oral microbiota have the potential to support novel probiotic approaches to improve nitrate metabolism in COPD patients.

Flow mediated dilatation to assess endothelial function

Flow mediated dilatation is non-invasive method of assessing endothelial function and assessing atherosclerosis in patients who are at high risk. NO is a known activator of guanylate cyclase which synthesises cyclic guanosine monophosphate (cGMP) from guanosine triphosphate (cGTP) leading to the relaxation of vascular smooth muscle. Thus not surprisingly the administration of NO_3^- has been correlated with reduced systolic and diastolic blood pressure[51,52] and potential cardioprotective effects.

Endothelial function can be assessed using the Endopat device which measures fingertip vascular response to brachial artery occlusion and then release after a period without blood flow.

1.2 RATIONALE FOR CURRENT STUDY

Dietary nitrate supplementation, in the form of beetroot juice, has a number of potentially advantageous effects in COPD. These include improving the response to pulmonary rehabilitation programme, making muscle contraction more efficient so it uses less oxygen, and improving how far people with low oxygen levels because of their lung disease can walk.

Although COPD is a lung disease, people with the condition are also at a higher risk of heart disease and stroke. There is also some evidence that beetroot juice can reduce blood pressure, but studies so far have been short term. A nutritional treatment that could produce a lasting reduction in blood pressure would be appealing, especially if it also improves people's ability to exercise.

Thus the PICOT research question is whether in people with stable COPD, oral dietary nitrate supplementation in the form of daily 70ml beetroot shot compared to a placebo drink of nitrate-depleted beetroot juice, reduces blood pressure over a three month period.

Additional outcomes will be effect on six minute walk distance, endothelial function, platelet aggregation, blood nitrate levels, plasma concentration of arginine/asymmetric dimethylarginine (ADMA) levels), and Fractional exhaled NO (FeNO).

We will also perform oral microbiome sampling.

2. STUDY OBJECTIVES

The purpose of this study is to investigate the effects of prolonged administration of beetroot (BR) juice versus placebo beverage ingestion on cardiovascular risk markers in COPD patients.

The following hypotheses are to be tested:

1) BRJ will reduce BP
2) BRJ will increase exercise capacity (6MWD)
3) BRJ will improve health related quality of life (CAT score)
4) BRJ will improve endothelial function - assessed by Endopat score
5) BRJ will improve markers of platelet activation (platelet-monocyte aggregates)
6) BRJ will alter ratio of plasma concentration of arginine/asymmetric dimethylarginine (ADMA)
7) BRJ will reduce serum BNP levels.
8) BRJ will increase fractional exhaled NO (FeNO)
9) BRJ will increase blood nitrate and nitrite levels
10) BRJ will alter the oral microbiome
11) BRJ will be an acceptable treatment (>80% participants comply with treatment defined as >80% doses consumed)

3. STUDY DESIGN

The study is a double-blind parallel group placebo-controlled trial to investigate the impact of dietary nitrate supplementation on BP. During the 3 month active treatment period 72 participants with stable COPD will consume 70mls beetroot juice each morning. This will either be active or placebo with the nitrate removed.

3.1 STUDY OUTCOME MEASURES

Primary endpoint Change in home monitored BP.

Secondary endpoints

i) Exercise capacity (6 minute walk test distance)
ii) Health related quality of life (HRQoL)

Exploratory endpoints

i) Endothelial function (Endopat score)
ii) Blood Brain Natriuretic Peptide levels (BNP)
iii) Markers of platelet activation (platelet-monocyte aggregates)
iv) Plasma concentration of arginine/asymmetric dimethylarginine (ADMA) levels)
v) Fractional exhaled NO (FeNO)
vi) Blood nitrate and nitrite levels
vii) Oral microbiome sampling
viii) Acceptability of treatment (>80% participants comply with treatment defined as >80% doses consumed)

4. PARTICIPANT ENTRY

4.1 PRE-RANDOMISATION EVALUATIONS

Patients will have a clinical diagnosis of COPD based on symptoms and spirometry.

We will identify COPD patients in four ways:

(i) opportunistically as they come into contact with the primary and secondary care teams – community clinics, secondary care clinics, 6 month post pulmonary rehabilitation reviews
(ii) using existing research/audit registers of patients including those who have completed pulmonary rehabilitation
(iii) by advertisement (local newspapers and through the BLF network of Breathe Easy Groups
(iv) using the NIHR UK Clinical Trials Gateway to contact suitable patients who have consented to be contacted about research.

4.2 INCLUSION CRITERIA

- Adult patients (>21 yrs) with stable COPD GOLD I-IV
- Established on stable pharmacotherapy for COPD
- Systolic blood pressure ≥130 mmHg recorded in clinic previously

4.3 EXCLUSION CRITERIA

- Unable to provide informed consent.
- AECOPD in the preceding month
- Significant comorbidity limiting exercise tolerance
- Significant comorbidity limiting life expectancy to <1 year
- Significant renal impairment (estimated glomerular filtration rate (eGFR) <30 ml.min1)
- Use of >3 blood pressure lowering medications
- Change in medication in the previous month
- Oral nitrate medication
- Current (in the last month) use of Beet Shots.

4.4 INITIAL ASSESSMENT FOR ELIGIBILITY

Where face to face visits are not possible, individuals who agree to be contacted will be asked to read the patient information sheet and then an initial telephone or video call will be made to go through eligibility criteria. If they agree to take part they will be asked to sign two copies of the consent form and return one.

They will then be sent a home BP monitor by courier.

4.5 WITHDRAWAL CRITERIA

No early stopping criteria are anticipated.

Data from patients who wish to be withdrawn from the study will be retained and used for analysis unless they request otherwise.

5. RANDOMISATION AND ENROLMENT PROCEDURE

5.1 RANDOMISATION OR REGISTRATION PRACTICALITIES

Patients will be asked to provide written informed consent. Once baseline measures have been completed participants will be randomised 1:1 using an online system (from SealedEnvelope™). They will receive a code and be given the matched beetroot shots (active or placebo) to take.

5.2 UNBLINDING

No circumstances where unblinding would be needed are anticipated.

6. TREATMENTS

6.1 TREATMENT ARMS

Active: 70mls Beet It Stamina shot from James White Ltd (6.5mmol Nitrate) taken orally each morning.
Placebo: 70mls Beet It Stamina placebo shot from James White Ltd (placebo with nitrate removed) taken orally each morning.

6.2 INTERACTION WITH OTHER DRUGS

No drug interactions are known.

6.3 DISPENSING AND ACCOUNTABILITY

Beetroot shots will be labelled with a code label linked to allocation i.e. active or placebo. They will be stored securely at room temperature and either given or sent to study participants by the trial team after randomization has occurred.

7. ADVERSE EVENTS

7.1 DEFINITIONS

Adverse Event (AE): any untoward medical occurrence in a patient or clinical study subject.
Serious Adverse Event (SAE): any untoward and unexpected medical occurrence or effect that:

- Results in death

- Is life-threatening – *refers to an event in which the subject was at risk of death at the time of the event; it does not refer to an event which hypothetically might have caused death if it were more severe*
- Requires hospitalisation, or prolongation of existing inpatients' hospitalisation
- Results in persistent or significant disability or incapacity
- Is a congenital anomaly or birth defect

Medical judgement should be exercised in deciding whether an AE is serious in other situations. Important AEs that are not immediately life-threatening or do not result in death or hospitalisation but may jeopardise the subject or may require intervention to prevent one of the other outcomes listed in the definition above, should also be considered serious.

7.2 REPORTING PROCEDURES
All adverse events should be reported. Depending on the nature of the event the reporting procedures below should be followed. Any questions concerning adverse event reporting should be directed to the Chief Investigator in the first instance.

7.3.1 Non serious AEs
All such events, whether expected or not, should be recorded.

7.3.2 Serious AEs
An SAE form should be completed and faxed to the Chief Investigator within 24 hours. However, relapse and death due to <condition>, and hospitalisations for elective treatment of a pre-existing condition do not need reporting as SAEs.
All SAEs should be reported to the <name of REC> where in the opinion of the Chief Investigator, the event was:

- 'related', ie resulted from the administration of any of the research procedures; and
- 'unexpected', ie an event that is not listed in the protocol as an expected occurrence

Reports of related and unexpected SAEs should be submitted within 15 days of the Chief Investigator becoming aware of the event, using the NRES SAE form for non-IMP studies. The Chief Investigator must also notify the Sponsor of all SAEs.
Local investigators should report any SAEs as required by their Local Research Ethics Committee, Sponsor and/or Research & Development Office.

Contact details for reporting SAEs
Fax: 020 73497778, attention Dr NS Hopkinson Please send SAE forms to:
Dr NS Hopkinson,
Royal Brompton Hospital,
Fulham Rd,
London SW3 6NP
SAE's should also be reported to jrco@imperial.ac.uk

8. ASSESSMENT AND FOLLOW-UP

See reference diagram and appendix for table.

(A) Where face to face visits are possible the procedure will be as follows:

Visit 1 – face to face

Eligibility for the study will be reviewed and if the patient wishes to participate they will sign a consent form. The following measures will be taken:

- Smoking status
- Current medication
- Other medical diagnoses
- Use of mouthwash (which can effect oral microbiota)
- Exacerbation history
- ECG
- Height and weight for BMI calculation
- CAT score completed
- Exhaled FeNO
- Blood drawn for
  - U&E, BNP,
  - Nitrate levels,
  - Platelet function
  - Plasma arginine/asymmetric dimethylarginine (ADMA) ratio
  - Oral samples for microbiome
- 6 minute walk test (two tests, the best value taken)

Subjects will be given Omron home BP monitor with instructions to take measurements for 4 days.

Visit 2

- Endothelial function test using the Endopat device
- Clinic BP
- Randomisation - 70ml BRJ-treated group or 70ml placebo-treated group

6 week follow-up phone call

Participants will receive a standard call encouraging them to drink all the assigned bottles, to keep their compliance diary and remind them to keep empty bottles.

11 week phone: reminder for self-activated 4 days home BP monitoring

Participants will receive a standard call encouraging them to drink all the assigned bottles, to keep their compliance diary and remind them to keep empty bottles. They will be asked to perform 4 days of home BP monitoring.

Visit 3

Post-intervention reassessment

Collect empty bottles for compliance count
Collect home BP monitor (4 days)
ECG CAT score FeNO test
Weight, and body mass index (BMI) measurement.
Clinic BP
Endothelial function
Blood tests (U&E, BNP, platelet function, nitrate levels)
Oral samples for microbiome
6 minute walk test
Collect diary

End of study

(B) Where face to face visits are not possible the procedure will be as follows

Visit 1 – Online

Subjects will have been sent Omron home BP monitor with instructions to take measurements for 4 days following the initial contact phone call to review eligibility

Visit 1 – face to face

Eligibility for the study will be reviewed and if the patient wishes to participate they will sign a consent form. The following measures will be taken:

- Smoking status
- Current medication
- Other medical diagnoses
- Use of mouthwash (which can effect oral microbiota)
- Exacerbation history
- ECG
- Height and weight for BMI calculation
- CAT score completed
- Exhaled FeNO
- Blood drawn for
  - U&E, BNP,
  - Nitrate levels,
  - Platelet function
  - Plasma arginine/asymmetric dimethylarginine (ADMA) ratio
  - Oral samples for microbiome
- 6 minute walk test (two tests, the best value taken)

Subjects will be given Omron home BP monitor with instructions to take measurements for 4 days.

Visit 2

- Endothelial function test using the Endopat device
- Clinic BP
- Randomisation - 70ml BRJ-treated group or 70ml placebo-treated group

6 week follow-up phone call

Participants will receive a standard call encouraging them to drink all the assigned bottles, to keep their compliance diary and remind them to keep empty bottles.

11 week phone: reminder for self-activated 4 days home BP monitoring!

Participants will receive a standard call encouraging them to drink all the assigned bottles, to keep their compliance diary and remind them to keep empty bottles. They will be asked to perform 4 days of home BP monitoring.

Visit 3

Post-intervention reassessment

Collect empty bottles for compliance count
Collect home BP monitor (4 days)
ECG CAT score FeNO test
Weight, and body mass index (BMI) measurement.
Clinic BP
Endothelial function
Blood tests (U&E, BNP, platelet function, nitrate levels)
Oral samples for microbiome
6 minute walk test
Collect diary

End of study

8.1 DETAILS OF ASSESSMENTS

Office blood pressure: [used for eligibility assessment] this will be performed, in accordance with the British Hypertension Society's standard operating procedure, using an automated cuff device after subject has been sat quietly for 5 minutes prior to measurement. The subject's arm will be supported.

Lung function: Spirometry will be performed according to ATS/ERS guidelines, taking the best of at least 3 efforts performed seated.

ECG: a twelve lead ECG will be performed after 5 minutes rest on a couch. Rhythm, heart rate, axis and QTc interval will be recorded.

FeNO: Exhaled nitric oxide (FeNO): We will use the NIOX VERO® device. The measurement of FeNO requires the participant to exhale slowly into a mouthpiece. The exhaled NO value is measured in parts per billion (PPB).

Anthropometric measurements: subjects' height (cm) will be measured without shoes with a wall mounted measure, and we will measure their weight (kg) using standardised scales.

Health status questionnaires: The COPD assessment (CAT) score will be administered to evaluate quality of life – this 8 item symptom score has been shown to be responsive both to exacerbations and to pulmonary rehabilitation.[63,64] Each item is scored 0-5 giving a score from 0 to 40 with 40 being the worst possible health status.

Blood sampling: Blood samples (a total of 20mls) will be drawn at baseline and again after 12 wks of treatment.

- **Analysis of nitrite and nitrate**: at visit 1 and 3 we will collect venous blood sample (3 mL) from subjects who for assessment of nitrite and nitrate levels using a chemoluminescence technique[53]. One of the research team will withdraw the blood samples through a 21-gauge butterfly needle inserted into an antecubital vein of the subjects. Blood samples will be collected in the morning between 8 and 11 am.

- Then, we will rapidly separate the plasma from red cells using a bench-top centrifuge for 3 min at 5,000 rpm. Plasma (1 mL) free of hemolysis is transferred into dark colored microtubes that also contain 6.5 mM *N*-ethylmaleimide (NEM) (100 mM; 6.5 µL) and 0.1 mM diethylenetriaminepentaacetic acid (DTPA) (10 mM; 10 µL). NEM and DTPA prevent the destruction of plasma S-nitrosothiols to NO_2^-. The samples are immediately frozen on dry ice and stored at −80 °C. In the lab, samples nitrite and nitrate will be first reduced to NO, which was then quantified using a NO analyser (NOA 280, Sievers). To determine total nitrite and nitrate concentrations, collectively termed 'NOx', samples will be added to 0.1 Mol/L vanadium (III) chloride in 1 M hydrochloric acid refluxing at 90°C under nitrogen. Nitrite concentrations will be then determined by addition of samples to 1.5 % potassium iodide in glacial acetic acid under nitrogen at room temperature. Finally, concentrations of nitrate will be calculated by subtraction of nitrite from NOx values[54].
  Empty tubes will also be collected in each batch to allow for background nitrate levels to be measured.

- **Analysis of brain natriuretic peptide (BNP)**: is a hormone secreted predominantly by the myocytes of the left ventricle in response to elevated wall stress. It has been shown to be a reliable diagnostic and prognostic tool in the management of congestive heart failure[55]. Recent studies show that BNP acts on the endothelium, for example, in mediating vasodilation through nitric oxide (NO),[56] possibly by influencing the activity of endothelial and inducible NO synthases (eNOS and iNOS, respectively), providing a link between natriuretic peptides and endothelial function.

- **Analysis of platelet aggregation:** we will study platelet function in trial participants to see whether nitrate supplementation attenuates ex vivo platelet aggregation in response to collagen and ADP. Blood will be drawn through a butterfly needle inserted into an antecubital vein of the subjects. Blood samples will be collected in the morning between 8 and 11 am and stored for analysis by PFA-100 in the lab. The PFA-100® (Dade Behring, Düdingen, Switzerland) is composed of a microprocessor-controlled device and single-use test cartridges[57]. The test cartridges consist of a sample reservoir, a capillary and a membrane coated with 2 mg equine type I collagen and either 10 mg epinephrine bitartrate (EPI cartridge) or 50 mg adenosine 5'- diphosphate (ADP cartridge). Blood is pipetted into the reservoir and aspirated through a capillary with a diameter of 200 mm with constant negative pressure resulting in high shear forces (5000–6000 s-1). The capillary ends in a membrane aperture with a diameter of 150 mm. Platelets adhere at the aperture where they are activated by the collagen and then aggregate. The two agonists epinephrine and ADP enhance aggregation. Finally, a platelet plug occludes the aperture and blood flow stops. The time measured in seconds from the beginning of the test until formation of an occluding platelet plug is called closure time (CT). If an occluding platelet plug does not form after 300 s, the analysis is stopped[58].

- **Analysis of plasma Arginine/Asymmetric dimethylarginine** (**ADMA)**: studying biochemical variables involved in the NO system such as Arginine and ADMA help us understand underling mechanisms of the effect of BRJ. ADMA is the endogenous inhibitor of the nitric oxide synthase (NOS) system. Arginine (Arg) is the substrate of NOS competing with ADMA. A low Arg/ADMA ratio is associated with the presence of vascular disease or endothelial dysfunction[59]. 5 mL venous blood taken into tubes by venipuncture through a butterfly needle. To obtain the serum, a member of the research team will collect the samples and centrifuged at 3500 rpm for 10 minutes. Then, samples will be stored at -80C till the analyses. Serum arginine and ADMA levels will be measured by HPLC (High-Performance Liquid Chromatography) using

commercial kits (EUREKA srl- Laboratory Division, Chiaravalle, Italy, cat no: Z58010) with a programmable fluorescence detector. Results will be given in lmol/L[60].

The 6-minute walk test distance (6MWD): we will be perform the 6MWD in an enclosed flat corridor, 30-m in length, following the American Thoracic Society guidelines[61]. Patients will be instructed to cover the longest distance possible in 6 minutes, with or without pause. During the test, standardized encouragement will be given to patients. The test will be performed twice and the greater distance will be recorded for pre- and post-intervention. Participants will wear the McRoberts MoveTest device during the test.
Oxygen saturation (SpO2) and heart rate will be monitored throughout the test using a pulse oximeter. During the test, a decrease in oxygen saturation level of equal to or less than 88% will be accepted as desaturation. The following parameters, including heart rate and oxygen saturation levels before and after the 6-minute walking test, the distance patients walk over 6 minutes, and the lowest and highest oxygen saturation levels during the test will be recorded during 6-minute walk distance test.

Analysis of oral Microbiota: Mouth swab will be taken and evaluated using 16S rRNA gene sequencing of total extracted saliva DNA on an Illumina MiSeq and analysed using an established pipeline in the section of Genomic Medicine. Comparison of microbial communities over time within patients and correlation with nitrate/nitrite levels across patients will allow identification of candidate organisms responsible for denitrification of nitrate to nitrite and NO. In addition, RT qPCR of functional genes (e.g. *nirK*, *nirS* and *narG*) in total extracted saliva RNA will demonstrate the expression of relevant microbial denitrification genes in response to nitrate.

Home self-activated BP monitoring: Participants will be given an Omron device to take home. They will be shown how to use it to measure their blood pressure and instructed to sit quietly for 5 minutes before they do so. They will be asked to take two readings (a minute apart) in the morning, the afternoon and the evening. They will be asked to do this for 4 days. The first day of readings will be discarded and the average of readings on days 2 to 4 taken.
In the event of a failure to do this at visit two they will be asked to repeat this before randomisation. In the event of a failure to do this before visit 3, the “clinic” blood pressure measure will be used for the primary endpoint.

Visit 2

Analysis of flow mediated dilatation: This will be measured using the EndoPAT-RHI system (Itamar Medical) which is an FDA-cleared non-invasive assessment of endothelial dysfunction. Measurements will be carried out in the non-dominant arm after a 20-min rest in the supine position in a quiet, temperature-controlled room (22–24°C). The EndoPAT test measure changes in vascular tone using sensors placed on the fingertips. These changes in arterial tone are elicited by creating a down-stream hyperemic response induced following a standard 5-minute occlusion of the brachial artery using a blood pressure cuff. Measurements from the contra-lateral arm are used to control for concurrent non-endothelial dependent changes in vascular tone to derive an index of endothelial function.

8.2 LOSS TO FOLLOW-UP

Subjects lost to follow up will be excluded from analysis.

8.3 TRIAL CLOSURE

The end of the trial is defined as the last patient's last visit.

9. STATISTICS AND DATA ANALYSIS

The study is powered for the primary outcome measure of change in home systolic BP response at 12 wk from baseline. Data from the ON-EPIC trial showed a fall of an (mean ± SD) 5 ± 3.7mmHg. Taking a power of 90% and significance level of 0.05, we would require 32 patients within each group to identify a 3mmHg fall with treatment. Allowing for a 10% dropout rate we will recruit 72 patients in total.

For the exploratory outcomes (blood tests, microbiome, endothelial function), will be analysed in at least 30 subjects. This is based on the number which produced a positive result in our previous trial (ON-EPIC) which investigated the effect of nitrate supplementation and pulmonary rehabilitation impact on flow mediated dilatation. Additionally, a previous study estimated of an expected increase in FMD of 25% from 5% to 6.7% after 6 weeks of beetroot juice intake in healthy volunteers, a sample size of 20 was needed to detect a difference with a power of 0.8 at significance level of α = 0.05 [assuming an SD of ultrasound FMD of 2.5] for a within-group comparison with baseline[52].

We will analyse all data using IBM statistical package for the social sciences (SPSS) software. For each group of the study, we will summarise baseline demographic and clinical variables.

We will use ANCOVA to compare blood pressure and other continuous variables between study groups, adjusting for baseline values. We will express all data, as mean ± SEM unless otherwise stated. In all cases, $P<0.05$ is considered statistically significant.

Data and all appropriate documentation will be stored for a minimum of 10 years after the completion of the study, including the follow-up period.

10. MONITORING

10.1 RISK ASSESSMENT

The study involves the use of a nutritional supplement and is considered to be low risk.

10.2 MONITORING AT STUDY COORDINATION CENTRE

Data will be collected on a paper CRF and then collected into a custom Excel database and analysed using SPSS. The study will be subject to audit by the Imperial College Joint Research Office.

11. REGULATORY ISSUES

11.1 ETHICS APPROVAL

The Study Coordination Centre has obtained approval from the London - West London & GTAC Research Ethics Committee (REC) and Health Regulatory Authority (HRA). The study must also receive confirmation of capacity and capability from each participating NHS Trust before accepting participants into the study or any research activity is carried out. The study will be conducted in accordance with the recommendations for physicians involved in

research on human subjects adopted by the 18th World Medical Assembly, Helsinki 1964 and later revisions.

11.3 CONSENT

Consent to enter the study must be sought from each participant only after a full explanation has been given, an information leaflet offered and time allowed for consideration. Signed participant consent should be obtained. The right of the participant to refuse to participate without giving reasons must be respected. After the participant has entered the trial the clinician remains free to give alternative treatment to that specified in the protocol at any stage if he/she feels it is in the participant's best interest, but the reasons for doing so should be recorded. In these cases the participants remain within the study for the purposes of follow-up and data analysis. All participants are free to withdraw at any time from the protocol treatment without giving reasons and without prejudicing further treatment.

11.4 CONFIDENTIALITY

Participants' identification data will be required for the registration process. The Study Coordination Centre will preserve the confidentiality of participants taking part in the study and is registered under the Data Protection Act.

11.5 INDEMNITY

Imperial College London holds negligent harm and non-negligent harm insurance policies which apply to this study.

11.6 SPONSOR

Imperial College London will act as the main Sponsor for this study. Delegated responsibilities will be assigned to the NHS trusts taking part in this study.

11.7 FUNDING

The Saudi Cultural Bureau are funding this study.

11.8 AUDITS AND INSPECTIONS

The study may be subject to inspection and audit by Imperial College London under their remit as Sponsor, the Study Coordination Centre and other regulatory bodies to ensure adherence to GCP.

12. TRIAL MANAGEMENT

A Trial Management Group (TMG) will be appointed and will be responsible for overseeing the progress of the trial. The day-to-day management of the trial will be co-ordinated through the Royal Brompton Study Coordination Centre, led by Ali Alasmari.

13. PUBLICATION POLICY

All publications and presentations relating to the study will be authorised by the Trial Management Group. The first publication of the trial results will be in the name of the Trial Management Group, if this does not conflict with the journal's policy. If there are named authors, these will include at least the trial's Chief Investigator, Statistician and Trial Coordinator. Members of the TMG and the Data Monitoring Committee will be listed and contributors will be cited by name if published in a journal where this does not conflict with the journal's policy. Authorship of parallel studies initiated outside of the Trial Management Group will be according to the individuals involved in the project but must acknowledge the contribution of the Trial Management Group and the Study Coordination Centre.

14. REFERENCES

1 Barnett K, Mercer SW, Norbury M, et al. Epidemiology of multimorbidity and implications for health care, research, and medical education: a cross-sectional study. *The Lancet* 2012; 380:37-43

2 Curtis KJ, O'Brien KA, Tanner RJ, et al. Acute Dietary Nitrate Supplementation and Exercise Performance in COPD: A Double-Blind, Placebo-Controlled, Randomised Controlled Pilot Study. *PLoS One* 2015; 10:e0144504

3 Sin DD, Anthonisen NR, Soriano JB, et al. Mortality in COPD: role of comorbidities. *European Respiratory Journal* 2006; 28:1245-1257

4 Calverley PMA, Anderson JA, Celli B, et al. Salmeterol and Fluticasone Propionate and Survival in Chronic Obstructive Pulmonary Disease. *New England Journal of Medicine* 2007; 356:775-789

5 Feary JR, Rodrigues LC, Smith CJ, et al. Prevalence of major comorbidities in subjects with COPD and incidence of myocardial infarction and stroke: a comprehensive analysis using data from primary care. *Thorax* 2010; 65:956-962

6 Rothnie KJ, Smeeth L, Pearce N, et al. Predicting mortality after acute coronary syndromes in people with chronic obstructive pulmonary disease. *Heart* 2016; 102:1442-1448

7 Hobbs DA, Kaffa N, George TW, et al. Blood pressure-lowering effects of beetroot juice and novel beetroot-enriched bread products in normotensive male subjects. *British Journal of Nutrition* 2012; 108:2066-2074

8 Kapil V, Khambata RS, Robertson A, et al. Dietary Nitrate Provides Sustained Blood Pressure Lowering in Hypertensive Patients - A Randomized, Phase 2, Double-Blind, Placebo-Controlled Study. *Hypertension* 2015; 65:320-327

9 d'El-Rei J, Cunha AR, Trindade M, et al. Beneficial Effects of Dietary Nitrate on Endothelial Function and Blood Pressure Levels. *International Journal of Hypertension* 2016; 2016:6791519

10 Siervo M, Lara J, Ogbonmwan I, et al. Inorganic Nitrate and Beetroot Juice Supplementation Reduces Blood Pressure in Adults: A Systematic Review and Meta-Analysis. *The Journal of Nutrition* 2013; 143:818-826

11 Monti LD, Barlassina C, Citterio L, et al. Endothelial Nitric Oxide Synthase Polymorphisms Are Associated With Type 2 Diabetes and the Insulin Resistance Syndrome. *Diabetes* 2003; 52:1270-1275

12 McNally B, Griffin JL, Roberts LD. Dietary inorganic nitrate: From villain to hero in metabolic disease? *Mol Nutr Food Res* 2016; 60:67-78

13 Alderton WK, Cooper CE, Knowles RG. Nitric oxide synthases: structure, function and inhibition. *Biochem J* 2001; 357:593-615

14 Furchgott RF, Zawadzki JV. The obligatory role of endothelial cells in the relaxation of arterial smooth muscle by acetylcholine. *Nature* 1980; 288:373-376

15 Ignarro LJ, Buga GM, Wood KS, et al. Endothelium-derived relaxing factor produced and released from artery and vein is nitric oxide. *Proc Natl Acad Sci U S A* 1987; 84:9265-9269

16 Ysart G, Miller P, Barrett G, et al. Dietary exposures to nitrate in the UK. *Food Addit Contam* 1999; 16:521-532

17 Lundberg JO, Weitzberg E. NO generation from inorganic nitrate and nitrite: Role in physiology, nutrition and therapeutics. *Arch Pharm Res* 2009; 32:1119-1126

18 Lundberg JO, Weitzberg E. NO-synthase independent NO generation in mammals. *Biochem Biophys Res Commun* 2010; 396:39-45

19 Lundberg JO, Govoni M. Inorganic nitrate is a possible source for systemic generation of nitric oxide. *Free Radic Biol Med* 2004; 37:395-400

20 Duncan C, Dougall H, Johnston P, et al. Chemical generation of nitric oxide in the mouth from the enterosalivary circulation of dietary nitrate. *Nat Med* 1995; 1:546-551

21 Benjamin N, O'Driscoll F, Dougall H, et al. Stomach NO synthesis. *Nature* 1994; 368:502
22 Lundberg JO, Weitzberg E, Cole JA, et al. Nitrate, bacteria and human health. *Nat Rev Microbiol* 2004; 2:593-602
23 Lundberg JO, Weitzberg E, Lundberg JM, et al. Intragastric nitric oxide production in humans: measurements in expelled air. *Gut* 1994; 35:1543-1546
24 Govoni M, Jansson EA, Weitzberg E, et al. The increase in plasma nitrite after a dietary nitrate load is markedly attenuated by an antibacterial mouthwash. *Nitric Oxide* 2008; 19:333-337
25 Bailey SJ, Fulford J, Vanhatalo A, et al. Dietary nitrate supplementation enhances muscle contractile efficiency during knee-extensor exercise in humans. *J Appl Physiol* 2010; 109:135-148
26 Richardson RS, Leigh JS, Wagner PD, et al. Cellular PO2 as a determinant of maximal mitochondrial O(2) consumption in trained human skeletal muscle. *J Appl Physiol* 1999; 87:325-331
27 Stuehr DJ, Santolini J, Wang ZQ, et al. Update on mechanism and catalytic regulation in the NO synthases. *J Biol Chem* 2004; 279:36167-36170
28 Bryan NS, Calvert JW, Gundewar S, et al. Dietary nitrite restores NO homeostasis and is cardioprotective in endothelial nitric oxide synthase-deficient mice. *Free Radic Biol Med* 2008; 45:468-474
29 Carlstrom M, Larsen FJ, Nystrom T, et al. Dietary inorganic nitrate reverses features of metabolic syndrome in endothelial nitric oxide synthase-deficient mice. *Proc Natl Acad Sci U S A* 2010; 107:17716-17720
30 Lundberg JO, Larsen FJ, Weitzberg E. Supplementation with nitrate and nitrite salts in exercise: a word of caution. *J Appl Physiol* 2011; 111:616-617
31 Jones AM, Bailey SJ, Vanhatalo A, et al. Reply to Lundberg, Larsen, and Weitzberg. *J Appl Physiol* 2011; 111:619
32 Lauer T, Heiss C, Balzer J, et al. Age-dependent endothelial dysfunction is associated with failure to increase plasma nitrite in response to exercise. *Basic Res Cardiol* 2008; 103:291-297
33 Stamler JS, Meissner G. Physiology of nitric oxide in skeletal muscle. *Physiol Rev* 2001; 81:209-237
34 Larsen FJ, Weitzberg E, Lundberg JO, et al. Effects of dietary nitrate on oxygen cost during exercise. *Acta Physiol (Oxf)* 2007; 191:59-66
35 Bailey SJ, Winyard P, Vanhatalo A, et al. Dietary nitrate supplementation reduces the O2 cost of low-intensity exercise and enhances tolerance to high-intensity exercise in humans. *J Appl Physiol (1985)* 2009; 107:1144-1155
36 Rochester CL, Vogiatzis I, Holland AE, et al. An Official American Thoracic Society/European Respiratory Society Policy Statement: Enhancing Implementation, Use, and Delivery of Pulmonary Rehabilitation. *Am J Respir Crit Care Med* 2015; 192:1373-1386
37 Berry MJ, Justus NW, Hauser JI, et al. Dietary nitrate supplementation improves exercise performance and decreases blood pressure in COPD patients. *Nitric Oxide* 2015; 48:22-30
38 Kerley CP, Cahill K, Bolger K, et al. Dietary nitrate supplementation in COPD: an acute, double-blind, randomized, placebo-controlled, crossover trial. *Nitric Oxide* 2015; 44:105-111
39 Friis AL, Steenholt CB, Løkke A, et al. Dietary beetroot juice – effects on physical performance in COPD patients: a randomized controlled crossover trial. *International Journal of Chronic Obstructive Pulmonary Disease* 2017; 12:1765-1773
40 Curtis KJ, O'Brien KA, Tanner RJ, et al. Acute Dietary Nitrate Supplementation and Exercise Performance in COPD: A Double-Blind, Placebo-Controlled, Randomised Controlled Pilot Study. *PLoS ONE* 2015; 10:e0144504

41 Leong P, Basham JE, Yong T, et al. A double blind randomized placebo control crossover trial on the effect of dietary nitrate supplementation on exercise tolerance in stable moderate chronic obstructive pulmonary disease. *BMC Pulmonary Medicine* 2015; 15:52

42 Kerley CP, Cahill K, Bolger K, et al. Dietary nitrate supplementation in COPD: An acute, double-blind, randomized, placebo-controlled, crossover trial☆. *Nitric Oxide* 2015; 44:105-111

43 Shepherd AI, Wilkerson DP, Dobson L, et al. The effect of dietary nitrate supplementation on the oxygen cost of cycling, walking performance and resting blood pressure in individuals with chronic obstructive pulmonary disease: a double blind placebo controlled, randomised control trial. *Nitric Oxide* 2015; 48:31-37

44 Chaouat A, Bugnet A-S, Kadaoui N, et al. Severe Pulmonary Hypertension and Chronic Obstructive Pulmonary Disease. *Am. J. Respir. Crit. Care Med.* 2005; 172:189-194

45 Zhang R, Wang X-J, Zhang H-D, et al. Profiling nitric oxide metabolites in patients with idiopathic pulmonary arterial hypertension. *European Respiratory Journal* 2016; 48:1386-1395

46 Sampaio-Maia B, Monteiro-Silva F. Acquisition and maturation of oral microbiome throughout childhood: An update. *Dental research journal* 2014; 11:291-301

47 Kilian M, Chapple ILC, Hannig M, et al. The oral microbiome – an update for oral healthcare professionals. *Bdj* 2016; 221:657

48 Allison SD, Martiny JBH. Resistance, resilience, and redundancy in microbial communities. *Proceedings of the National Academy of Sciences* 2008; 105:11512-11519

49 Vanhatalo A, Blackwell JR, L'Heureux JE, et al. Nitrate-responsive oral microbiome modulates nitric oxide homeostasis and blood pressure in humans. *Free radical biology & medicine* 2018; 124:21-30

50 Wu J, Peters BA, Dominianni C, et al. Cigarette smoking and the oral microbiome in a large study of American adults. *The ISME journal* 2016; 10:2435-2446

51 Larsen FJ, Ekblom B, Sahlin K, et al. Effects of dietary nitrate on blood pressure in healthy volunteers. *N Engl J Med* 2006; 355:2792-2793

52 Webb AJ, Patel N, Loukogeorgakis S, et al. Acute blood pressure lowering, vasoprotective, and antiplatelet properties of dietary nitrate via bioconversion to nitrite. *Hypertension* 2008; 51:784-790

Appendix 1. Summary of investigations, treatment and assessments

	Visit 1	Visit 2	6 week call	12 week call	Visit 3
Review eligibility	X				
Informed consent	X				
ECG	X				X
CAT score	X				X
Spirometry	X				
FeNO	X				X
BMI	X				X
Blood tests	X				X
Oral samples for microbiome	X				X
U&E, BNP	X				X
6 minute walk test	X				X
Home BP monitoring given	X				X
Clinic BP		X			X
Endothelial function		X			X
Phone call to check compliance			X		
Phone call to remind to do home BP monitoring				X	
