# Supplementary material for: Oral nitrate supplementation improves cardiovascular risk markers in COPD: ON-BC, a randomised controlled trial
Source: Eur Respir J. 2024 Feb 1;63(2):2202353. doi: 10.1183/13993003.02353-2022 (PMC10831142; doi:10.1183/13993003.02353-2022)
Supplement: Supplementary file 3 [file ERJ-02353-2022.Shareable.pdf]

EUROPEAN RESPIRATORY JOURNAL
ORIGINAL RESEARCH ARTICLE
A.M. ALASMARI ET AL.

SHAREABLE PDF

Oral nitrate supplementation improves cardiovascular risk markers in COPD: ON-BC, a randomised controlled trial

Ali M. Alasmari[1,2], Abdullah S. Alsulayyim[1,3], Saeed M. Alghamdi[4], Keir E.J. Philip[1,5], Sara C. Buttery[1], Winston A.S. Banya[1], Michael I. Polkey[1,5], Paul C. Armstrong[6], Matthew J. Rickman[7], Timothy D. Warner[6], Jane A. Mitchell[7] and Nicholas S. Hopkinson[1]

[1]National Heart and Lung Institute, Royal Brompton Campus, Imperial College London, London, UK. [2]Respiratory Therapy Department, College of Medical Rehabilitation Sciences, Taibah University, Madinah, Saudi Arabia. [3]Respiratory Therapy Department, Faculty of Applied Medical Sciences, Jazan University, Jazan, Saudi Arabia. [4]Clinical Technology Department, Umm Al-Qura University, Makkah, Saudi Arabia. [5]Respiratory Medicine, Royal Brompton and Harefield Hospitals, London, UK. [6]Centre for Immunobiology, Blizard Institute, Faculty of Medicine and Dentistry, Queen Mary University of London, London, UK. [7]National Heart and Lung Institute, Cardiothoracic Pharmacology, Vascular Biology, Imperial College London, London, UK.

Corresponding author: Nicholas S. Hopkinson (n.hopkinson@ic.ac.uk)

Check for updates

Shareable abstract (@ERSpublications)
The ON-BC trial shows that 12 weeks of oral nitrate supplementation, in the form of beetroot juice, lowers blood pressure, improves vascular function and increases exercise capacity in people with COPD https://bit.ly/3MAGiZM

Cite this article as: Alasmari AM, Alsulayyim AS, Alghamdi SM, *et al.* Oral nitrate supplementation improves cardiovascular risk markers in COPD: ON-BC, a randomised controlled trial. *Eur Respir J* 2024; 63: 2202353 [DOI: 10.1183/13993003.02353-2022].

This extracted version can be shared freely online.

Copyright ©The authors 2024.

This version is distributed under the terms of the Creative Commons Attribution Licence 4.0.

This article has an editorial commentary: https://doi.org/10.1183/13993003.02238-2023

Received: 6 Dec 2022
Accepted: 6 Nov 2023

Abstract

Background Short-term studies suggest that dietary nitrate (NO_3^-) supplementation may improve the cardiovascular risk profile, lowering blood pressure (BP) and enhancing endothelial function. It is not clear if these beneficial effects are sustained and whether they apply in people with COPD, who have a worse cardiovascular profile than those without COPD. Nitrate-rich beetroot juice (NR-BRJ) is a convenient dietary source of nitrate.

Methods The ON-BC trial was a randomised, double-blind, placebo-controlled parallel group study in stable COPD patients with home systolic BP (SBP) measurement ⩾130 mmHg. Participants were randomly allocated (1:1) using computer-generated, block randomisation to either 70 mL NR-BRJ (400 mg NO_3^-) (n=40) or an otherwise identical nitrate-depleted placebo juice (0 mg NO_3^-) (n=41), once daily for 12 weeks. The primary end-point was between-group change in home SBP measurement. Secondary outcomes included change in 6-min walk distance (6MWD) and measures of endothelial function (reactive hyperaemia index (RHI) and augmentation index normalised to a heart rate of 75 beats·min^{-1} (AIx75)) using an EndoPAT device. Plasma nitrate and platelet function were also measured.

Results Compared with placebo, active treatment lowered SBP (Hodges–Lehmann treatment effect −4.5 (95% CI −5.9– −3.0) mmHg), and improved 6MWD (30.0 (95% CI 15.7–44.2) m; p<0.001), RHI (0.34 (95% CI 0.03–0.63); p=0.03) and AIx75 (−7.61% (95% CI −14.3– −0.95%); p=0.026).

Conclusions In people with COPD, prolonged dietary nitrate supplementation in the form of beetroot juice produces a sustained reduction in BP, associated with an improvement in endothelial function and exercise capacity.
